# Navigating the Landscape of Dry Assembling Ordered Particle Structures: Can Solvents Become Obsolete?

**DOI:** 10.1002/smll.202405410

**Published:** 2024-09-16

**Authors:** Kai Sotthewes, Ignaas S. M. Jimidar

**Affiliations:** ^1^ Physics of Interfaces and Nanomaterials MESA+ Institute University of Twente P.O. Box 217 Enschede 7500AE The Netherlands; ^2^ Department of Chemical Engineering CHIS Vrije Universiteit Brussel Brussels 1050 Belgium; ^3^ Mesoscale Chemical Systems MESA+ Institute University of Twente P.O. Box 217 Enschede 7500AE The Netherlands

**Keywords:** colloidal particles, crystals, dry assembly, monolayers, surface interaction forces

## Abstract

A spur on miniaturized devices led scientists to unravel the fundamental aspects of micro‐ and nanoparticle assembly to engineer large structures. Primarily, attention is given to wet assembly methods, whereas assembly approaches in which solvents are avoided are scarce. The “dry assembly” strategies can overcome the intrinsic disadvantages that are associated with wet assembly, e.g., the lack of versatility and scalability. This review uniquely summarizes the recent progress made to create highly ordered particle arrays without using a wet environment. Before delving into these methods, the surface interactions (e.g., van der Waals, contact mechanics, capillary, and electrostatics) are elaborated, as a profound understanding and balancing these are a critical aspect of dry assembly. To manipulate these interactions, strategies involving different forces, e.g., mechanical‐based, electrical‐based, or laser‐induced, sometimes in conjunction with pre‐templated substrates, are employed to attain ordered colloidal structures. The utilization of the ordered structures obtained without solvents is accompanied by specific examples. Dry assembly methods can aid us in achieving more sustainable assembly processes. Overall, this Review aims to provide an easily accessible resource and inspire researchers, including novices, to broaden dry assembly horizons significantly and close the remaining knowledge gap in the physical phenomena involved in this area.

## Graphical abstract

This unique review critically elaborates on the recently proposed assembly of colloids into ordered structures without using solvents, i.e., dry assembly methods. Additionally, challenges and opportunities are required to inspire scientists to advance the field from a fundamental and application perspective.

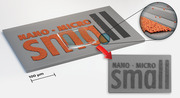

## 1 Introduction

**Figure 1:**
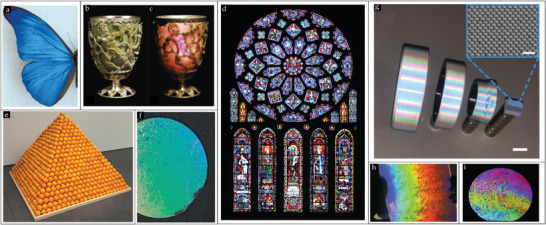
a)Real color image of the blue iridescence from a Morpho rhetenor wing. Reproduced with permission.^[^ ^12^ ^]^ Copyright 2003, Springer Nature. The Lycurgus Cup at the British Museum in b) reflected and c) transmitted mode due to the formation of Ag–Au alloy NPs in glass. (b,c) Reproduced with permission.^[^ ^261^ ^]^ Copyright 2007, Springer Nature. d) The stained glass North Rose window of the Chartres Cathedral, France, 1190–1220 CE. Reproduced with permission.^[^ ^262^ ^]^ Copyright 2018, Mark Cartwright. e) An ordered pile of stacked oranges. Reproduced with permission.^[^ ^263^ ^]^ Copyright 2016, Don Steward. f) A monolayer of 1 µm colloids assembled on a wafer. Reproduced with permission.^[^ ^264^ ^]^ Copyright 2011, Wiley‐VCH. g) Assembled colloidal crystals on curved substrates. Obvious iridescence structure colors observed under the illumination of colloidal crystal films attained on the curved stainless steel metal ring surfaces and the quartz glass curved surface. (scale bar, 1 cm). The inset displays the SEM image of the respective colloidal film (scale bar, 2 µm). Reproduced with permission.^[^ ^228^ ^]^ Copyright 2022, Wiley‐VCH. h) Visible diffraction pattern from a single‐crystal monolayer of 1 µm‐diameter PS beads on a PDMS substrate. Reproduced with permission.^[^ ^99^ ^]^ Copyright 2014, Wiley‐VCH. i) Wafer carrying a bidisperse colloidal monolayer, of which the individual single‐crystal domains can be distinguished by their vivid coloration effects. Reproduced with permission.^[^ ^265^ ^]^ Copyright 2011, Wiley‐VCH.

Setting foot in the miniaturization era, scientists utilized micro‐ and nanoscale building blocks to craft functional (macroscopic) structures. The latter, in conjunction with Feynman's famous exclamation, “There's plenty of room at the bottom!,”^[^ ^1^ ^]^ manifested a widespread interest across disciplines to discover and reveal the secrets held by nature to create structures, e.g., the folding of proteins into 3D structures, the assembly of amphiphilic lipids into bilayer cell membranes,^[^ ^2^, ^3^ ^]^ and the ubiquitous presence of fascinating display of iridescence colors from birds and butterflies (cf. **Figure** **1**a) which captures the essence of visible light interactions with intricate nano‐ and microscale structures, elucidating the ingenuity present in nature to generate coloration effects.^[^ ^4^ ^]^ Such coloration optical effects were already being leveraged in Medieval times to stain glass with metal nanoparticles,^[^ ^5^, ^6^, ^7^ ^]^ e.g., the Roman Lycurgus cup (Figure 1b,c) or church windows (Figure 1d). Altogether, these examples inspired and fed the perpetual curiosity of scientists in the modern days to fundamentally unravel and concurrently replicate these vivid color effects at the macroscale by meticulously assembling structures using colloidal particles,^[^ ^4^, ^6^, ^8^, ^9^, ^10^, ^11^, ^12^, ^13^, ^14^ ^]^ as showcased in Figure 1e–g.

Therefore, the literature on colloidal assembly is vast, as many researchers have devoted their work spanning fundamental aspects as well as emerging application areas. From a fundamental perspective, colloidal particles serve as excellent model objects for smaller building blocks as atoms and molecules to understand condensed matter physics processes, e.g., nucleation, crystallization, and phase transitions.^[^ ^15^, ^16^ ^]^ On the application side of things, advances have not solely pertained to the coloration effects mentioned above in photonic crystals or pigments but extended over a wide breadth of domains, including colloidal nanosphere lithography,^[^ ^17^, ^18^, ^19^ ^]^ control of surface wetting properties, filtering, bio and chemical sensing,^[^ ^20^, ^21^, ^22^, ^23^ ^]^ anti‐reflective surfaces, smart windows,^[^ ^24^ ^]^ electrode materials, microelectronics, and robotics.^[^ ^25^, ^26^ ^]^ As such, the controlled assembly of colloidal particles into ordered structures can be simple and cost‐effective to produce, and thus appealing for industry. Moreover, these structures unlock the opportunity for producing smart sensors and devices as colloids are programmable to be highly responsive to external stimuli,^[^ ^24^ ^]^ efficient surface coatings,^[^ ^27^ ^]^ smart nanostructured materials for a plethora of applications,^[^ ^26^ ^]^ robust‐lightweight materials.^[^ ^8^ ^]^ Thus, these promising colloidal assembly‐based applications underpin their importance in our quest for sustainable, energy‐efficient, and low‐cost miniaturized devices for a more sustainable society.

The development of approaches to assemble colloidal particles into ordered 2D monolayers or 3D structures can be broadly characterized in wet and dry methods, i.e., assembly without using solvents. Comparatively, a plethora of wet assembly methods have been proposed, optimized, and refined over the last decades, whereas progress in dry assembly approaches is lacking. The large spectrum of wet assembly methods, including dip‐coating, electrodeposition, and convective assembly spin‐coating, are elaborately presented in excellent reviews that the interested reader is kindly referred to refs. [8, 25, 28, 29]. Despite all advances in wet assembly methods, their remaining Achilles' heel is that they massively hinge on solvent and particle properties. e.g., material and size, and concomitantly on highly optimized conditions, such as evaporation rate, temperature, and pH. Consequently, wet assembly methods suffer from the lack of versatility, large scalability, and reproducibility. In this regard, dry assembly methods are promising alternatives as they can be faster, more reproducible, have a higher tolerance than wet methods for process conditions, and apply to a broader spectrum of particles. However, they are a feat hardly undertaken to produce ordered particle structures. To this end, assembly methods in which the solvents are not utilized remain poorly understood, underscoring the remaining fundamental knowledge gap to achieve a quantum leap in the dry assembly of ordered colloidal structures.

For centuries long, humankind has mastered the architectural skills to “manually” position building blocks, such as tiles and bricks, to construct desired structures using adhesives. Furthermore, stacking of marbles or fruits (cf. Figure 1e) by hand is also commonplace. Therefore, it is, in fact, surprising that the dry assembly of ordered colloidal particle structures is still in its infancy, which can be attributed to the complexity of mutually present strong surface interaction forces among micro‐ and nanoparticles, rendering dry assembly processes daunting and complex. Thus, this exemplifies that building at the small micro‐ and nanoscale is incommensurate with the macroscale, as in contrast to the macroscale where we have access to individual building blocks, the particles have the propensity to form aggregates at the smaller scale due to the strong interaction forces. These strong interaction forces encompass the van der Waals forces, contact mechanics force, capillary forces, and electrostatic interactions, which can be circumvented when particles are dispersed in solvents, signifying why the scientific community has commonly shared their preference for utilizing wet assembly methods.

The colloidal dry assembly domain is fortunately not gloomy after all, as entering the micro‐ and nanotechnology realm has spurred the exploration of dry assembly methods that have proven to be faster than their wet counterparts. In contrast to wet assembly, typically based on self‐assembly processes, dry methods demand a driving force, e.g., mechanical, laser‐induced, or electrical, to direct the assembly of particles into ordered structures. Among the relatively few dry assembly methods reported so far, the rubbing of dry powder has recently garnered significant attention in many studies and applications due to its ease and simplicity in attaining conspicuously present monolayers or arrays over large areas, albeit mostly on soft elastomeric substrates. On the other hand, dry assembly methods to attain 3D‐ordered particle structures remain scarce, underpinning the opportunities ahead of us to mature the field.

This review commences with a concise qualitative discussion on the surface interaction forces involved in dry assembly to provide the necessary background to comprehend how these forces can mutually influence the assembly process, catering to a broad readership. This sets the stage for critically delineating the reported diversity of dry assembly methods based on various physical mechanisms in subsequent sections. The well‐established microcontact printing of ordered particle arrays in which wet and dry assembly strategies have been employed in complementary ways is also elaborately presented. Next, applications that utilized dry assembly methods are highlighted, and the review is concluded with an outlook section. As a dedicated review discussing the complexity and opportunities of dry assembly methods ordered particle structures is uncharted, we envision this review as a fertile ground to advance the field and inspire scientists to refine further existing or propose novel dry assembly approaches, including producing 3D‐ordered structures, and comprehend the physical phenomena in dry assembly to close the fundamental knowledge gap in this field.

## 2 Relevant Interaction Forces

**Figure 2:**
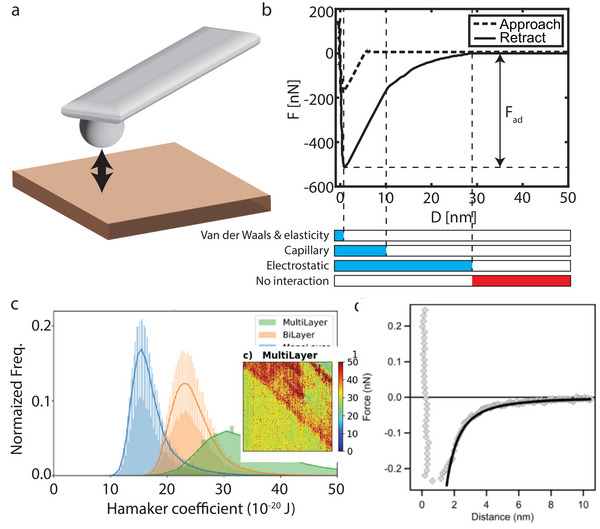
a) Schematic representation of force‐distance (*F*(*D*)) spectroscopy using a colloidal probe. An AFM cantilever is approached and retracted from the substrate while the force is monitored. b) Typical *F*(*D*)‐curve between a silica colloid and a glass substrate. The blue bars represent the relevant type of interaction force when the colloidal probe has been released at a distance (*D*) from the flat substrate, while the red bar signifies the range where all interactions vanish. Reproduced with permission.^[^ ^33^ ^]^ Copyright 2023 American Chemical Society. c) Distribution of the Hamaker coefficient measured with the bimodal AFM of monolayer, bilayer and trilayer graphene on copper. Inset: Hamaker constant map of trilayer graphene on copper. Reproduced with permission.^[^ ^51^ ^]^ Copyright 2018 American Chemical Society. d) Attractive van der Waals force between two neutral amidine latex particles at pH 5.6. Solid line is the best fit with the van der Waals attraction. Reproduced with permission.^[^ ^58^ ^]^ Copyright 2012 Chimia.

The (self‐)assembly of nano‐ and microparticles is determined by the forces interacting between the particles, and between the particle and the hosting surface.^[^ ^30^, ^31^ ^]^ In order to gain full control of the assembly process, it is of the utmost importance to know which interaction forces are present, their relevant length scales, and their interaction strength. Therefore, measurement techniques are needed that can detect these forces at the micro‐ and nanoscale for various systems. In this section, we will discuss the most important forces present when dry‐assembling micro‐ and nanoparticles and how we can measure and concurrently comprehend the contribution of these forces to design controlled assembly processes. One method that can detect the most important interaction forces at these length scales is atomic force microscopy (AFM) with all its derivatives. One of them that is especially suitable to detect interaction forces is the force‐distance (*F*(*D*)) mode in which the cantilever of the AFM is brought in and out of contact with the investigated surface (see **Figure** **2**a).^[^ ^32^, ^33^, ^34^, ^35^ ^]^ The cantilever geometry can be a cone, a pyramid, or a sphere, depending on the type of AFM tip. While the tip is approaching, in contact or retracting the surface, the force is recorded, revealing the interaction between the cantilever and the probed surface. An example of a *F*(*D*)‐measurement is shown in Figure 2b for a silica colloid on a glass substrate.^[^ ^33^ ^]^ When approaching, the probe jumps into contact with the substrate because the force gradient (mostly the van der Waals force) is larger than the effective elastic constant of the cantilever.^[^ ^32^, ^34^ ^]^ During the retraction phase, when the colloidal probe is released from the substrate, the colloid particle experiences different adhesion‐type forces, as indicated by the blue bars in Figure 2b. From this curve, the strength and relevant length scale can be extracted for the different interaction forces for the particular system. In this case, the adhesion force (*F*_ad_) is mainly composed of four forces: i) the van der Waals force, ii) the contact mechanics force, iii) the capillary force, and iv) the electrostatic force. In the remainder of this section, these relevant interaction forces are elaborated on in more detail.

### 2.1 Van Der Waals Force

#### 2.1.1 Description of the Van Der Waals Force

The van der Waals forces (*F*_vdW_) is an interatomic interaction force and stems from the electromagnetic interactions between neutral molecular dipoles. The van der Waals force is present in every situation and, thus, also in the assembly of particles. For rigid and surface homogeneous spheres, the van der Waals forces can be semi‐quantitatively described as refs. [36, 37, 38]

(1) $$\begin{array}{c} F_{\text{vdW}}=\frac{A_{H}R}{6D^{2}}\frac{R_{1}R_{2}}{R_{1}+R_{2}}=\frac{A_{H}R}{12D^{2}}\;\left(R_{1}=R_{2}=R\text{, for two identical spheres}\right) \end{array}$$

(2) $$\begin{array}{c} F_{\text{vdW}}=\frac{A_{H}R}{6D^{2}}\;(R=R_{2}\gg R_{1}\text{, for sphere and surface)} \end{array}$$

where *A*_H_ is the Hamaker constant, *R* is the particle radius and *D* the separation distance between the particles outermost surfaces. The values for the Hamaker constant usually range from [0.4 − 4] × 10^−19^ J. From Equations (1) and (2), it is immediately clear that the origin and magnitude of the van der Waals force are proportional to the particle size and decays quadratically with the distance between the particles.

#### 2.1.2 Experimental Determination of the Van Der Waals Force on the Microscale

The quantification of the van der Waals force between particles in practice is challenging because of the difficulty in defining the effective Hamaker constant and separation distance between heterogeneous, rough, and deformable particles. For the simplest geometry, two parallel plates separated by distance *D*, the van der Waals force was measured in the 50's^[^ ^39^, ^40^, ^41^, ^42^ ^]^ using a spring balance, which was later extended to sphere‐flat and crossed‐cylinder geometries. A good agreement between the experiments and theory was found for various material combinations.^[^ ^43^ ^]^

Although the spacing between the plates was already in the micrometer range, it was not until 1992 that the van der Waals interaction between a particle and a surface on the nanoscale was measured,^[^ ^44^ ^]^ albeit indirectly using a micron‐sized cavity. This is challenging due to the increasing influence of other forces, e.g., electrostatic and capillary interactions, and the decreasing strength of the van der Waals interaction. Sandoghar et al., achieved this using sodium atoms which were placed between two gold‐coated mirrors.^[^ ^44^ ^]^ A laser was used to excite the atoms, and subsequently, the excitation energy of the atom was measured as a function of the mirror‐separation distance. The observed trend was well explained with the van der Waals interaction model.

#### 2.1.3 Experimental Determination of the Van Der Waals Force on the Nanoscale

The first direct measurement of the van der Waals interaction between two atoms was performed only in 2013 by Béguin et al.,^[^ ^45^ ^]^ using two isolated Rydberg atoms (atoms with one electron in a highly excited state) locked in an optical trap that was separated by a controlled distance. The interaction between the atoms was measured, revealing the expected van der Waals behavior.

Using an AFM, the nanoscale adaptation of the previously mentioned spring balance, the interaction was measured between different organic molecules and a metallic surface.^[^ ^46^ ^]^ A linear growth in the van der Waals attraction was found with the molecular size, originating from the increased deconfinement of electrons in the molecules. A similar observation was made by Kawai and co‐workers^[^ ^47^ ^]^ for noble gas atoms. Using an Xe‐functionalized AFM tip, they found that even the adsorption geometry has an influence on the measured van der Waals force due to the adsorption‐induced charge redistribution.

For very flat and smooth surfaces, such as 2D materials, it is possible to determine the Hamaker constant and thus the van der Waals strength spatially resolved^[^ ^48^, ^49^ ^]^ using bimodal AFM, a method which uses the simultaneous excitation of the first two flexural eigenmodes of the cantilever. The phase shift in both of the eigenmodes is recorded and converted into a Hamaker constant.^[^ ^50^, ^51^ ^]^ An example is shown in Figure 2c, in which the Hamaker constant distribution is shown for mono‐, bi‐, and trilayer graphene on copper.^[^ ^51^ ^]^ A clear difference is observed between the three samples, although the top layer is a single carbon layer in all three of the cases, emphasizing the importance and influence of the supporting substrate on the van der Waals force. From experiments performed on MoS_2_ on Au, it was observed that the Hamaker constant remains constant for three or more MoS_2_ layers.^[^ ^49^ ^]^ At these thicknesses, the influence of the supporting substrate on the Hamaker constant vanishes. Also, within the layer, differences in the Hamaker constant are observed (see inset of Figure 2c) induced by the grain boundaries in the copper substrate. All the aforementioned examples underline the complexity, if not impossibility, in determining the van der Waals force on the nanoscale due to the influence of the (local) environment on the charge redistribution on the surface or particle.

#### 2.1.4 Evaluation of the Experimental Measured Van Der Waals Force

On the microscale, most of the research involves the van der Waals interaction between two colloidal particles or a colloid and another substrate.^[^ ^52^, ^53^, ^54^, ^55^ ^]^ However, a majority of these experiments are performed in solution. The reason is two‐fold: i) the force applied by the tip to the surface is reduced by a factor of 10–100 compared to the force in the air (or under dry conditions) due to the absence of the capillary force, enhancing the influence and detectability of other forces, and ii) for the many applications of particles dispersed in a solution.^[^ ^8^, ^25^, ^28^, ^29^, ^56^ ^]^

The colloidal probe method allows to investigate the interaction between a colloid attached to an AFM cantilever (cf. Figure 2a) and another colloid or a surface.^[^ ^56^, ^57^, ^58^ ^]^ Measuring the van der Waals force is easy as long as the system is electrically neutral because the Coulomb interaction is usually magnitudes stronger compared to the van der Waals interaction,^[^ ^33^ ^]^ masking the van der Waals contribution. This was, for instance, shown for two 3.3 µm amidine particles in a KCl solution with a pH of 5.6 (see Figure 2d). From the fit, a Hamaker constant of 4.5 × 10^−21^ J is obtained while calculations based on the Liftshitz theory predict a higher value of 9.0 × 10^−21^ J. This discrepancy is explained by the roughness of the particles, which reduces the van der Waals interactions. As can be seen in Figure 2d, the van der Waals interaction vanishes ≈10 nm, while the roughness of the 3.3 µm particle is in the same range. Hence, the van der Waals force heavily depends on the exact contact location, which is often unknown in the experiment. Due to the short interaction length and the relatively weak interaction strength compared to the other interaction forces present, the van der Waals force is difficult to measure in air environments and plays a minor role in the assembly of micro‐ and nano‐sized particles without solvents.^[^ ^33^ ^]^

### 2.2 Contact Mechanics Force

#### 2.2.1 Introduction to the Contact Mechanics Force

The moment two bodies are in contact, the contact mechanics force comes into play. This force is the result of the intrinsic deformation of solids in contact and can be divided into compressive and adhesive forces perpendicular to the interface and frictional forces in the tangential direction.^[^ ^59^ ^]^ For the assembly of particles, the frictional forces are, in general, relatively small due to the spherical shape of the particles. However, the adhesive forces perpendicular to the interface are one of the most dominant forces in the assembly of particles on the nano‐ and microscale. Although dominant, extracting the exact strength of the contact mechanics force at these length scales remains a challenge.

#### 2.2.2 The Hertz Model

The concept of contact mechanics was first introduced by Hertz in 1882,^[^ ^60^ ^]^ who found a solution to the contact problem between two elastic bodies with curved surfaces without adhesive forces. It is a description of the contact stress as a function of the normal contact force, the bending radii of the bodies, and their respective elastic moduli. When an elastic sphere indents an elastic plate, the displacement (or indentation) *d* leads to a contact radius (*a*) of

(3) $$a=\sqrt{Rd}$$

with *R* as the radius of the elastic sphere. The applied force (*F*_App_) is related to the displacement by

(4) $$F_{\text{App}}=\frac{\sqrt{R}d^{3/2}}{K}$$

with *K* the combined elastic modulus of the two bodies, given by $K=4/3{\left(\left(1-\nu_{t}^{2}\right)/Y_{t}\right)}^{-1}+{\left(\left(1-\nu_{s}^{2}\right)/Y_{s})\right)}^{-1}$ where *Y*_t_, ν_t_ *Y*_s_ and ν_s_ are the Poisson ratios and Young's moduli of tip (in this example a sphere) and sample, respectively. When Equations (3) and (4) are combined, the contact radius is related to the applied load by

(5) $$a=\sqrt[{3}]{RF_{\text{App}}K}$$

For the contact between two spheres with radii *R*_1_ and *R*_2_, the above equations still hold with the introduction of an effective radius *R*_e_ defined as $\frac{1}{R_{e}}=\frac{1}{R_{1}}+\frac{1}{R_{2}}$.

#### 2.2.3 The JKR, DMT and MD Description of the Contact Mechanics Force

The Hertz model is a good approximation of the contact mechanics force on the macroscopic scale. At the nanoscopic scale, the solid bodies can still be treated as a continuum, but the effects of surface forces in the immediate vicinity of the contact region become relevant.^[^ ^61^, ^62^ ^]^ The adhesive stress induced by the two bodies at the nanoscale is typically replaced by the Lennard–Jones potential. This was further developed by Bradley^[^ ^63^ ^]^ for two smooth rigid spheres in contact, and the contact force (*F*_con_) is given by

(6) $$F_{\text{con}}=2\pi w_{\text{adh}}R$$

where *w*_adh_ is the work of adhesion when two bodies in contact are separated (note: *R* is replaced with *R*_e_ in the case of two spheres). The work of adhesion is given by *w*_adh_ = Δγ = γ_1_ + γ_2_ − γ_1, 2_ with γ_1_, γ_2_andγ_1, 2_ the corresponding surface energies of the two spheres with the environment and with each other, respectively.

Two competing theories were simultaneously developed for two elastic spheres in contact at small scales. The Johnson–Kendall–Roberts (JKR)^[^ ^64^ ^]^ and the Derjaguin–Möller–Toporov (DMT)^[^ ^65^ ^]^ theories, which both included adhesion forces such as van der Waals but also capillary and electrostatic forces (all these interactions are represented by *w*_adh_). At first, the community regarded these two theories as competitive until Tabor^[^ ^66^ ^]^ pointed out that they are actually opposite extremes of the parameter μ:

(7) $$\mu =\sqrt[{3}]{\frac{Rw_{\text{adh}}^{2}K^{2}}{z_{0}^{3}}}$$

where *z*_0_ is the equilibrium separation, the distance at which attractive and repulsive forces between two bodies are balanced, resulting in a stable position. The parameter μ represents the magnitude of the elastic deformation compared with the range of surface forces. The JKR theory assumes that the adhesive forces are confined within the contact area and thus gives a contact force of *F*_con_ = 1.5π*w*_adh_ *R* (see Figure 5a). On the other hand, the DMT theory describes that adhesive forces act outside of the contact area and yields *F*_con_ = 2π*w*_adh_ *R* (similar to the result of Bradley,^[^ ^63^ ^]^ cf. Figure 5b).

**Figure 3:**
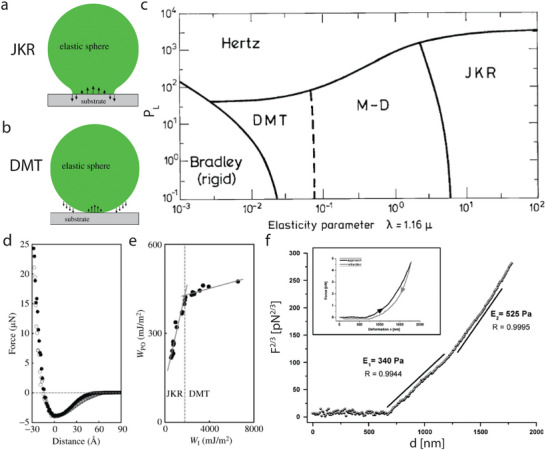
a,b) Sketch of traction distribution in the DMT and JKR theory. In DMT, the adhesive forces are confined outside the contact area, while both compressive and adhesive forces are exchanged in the contact area for the JKR model. Reproduced with permission.^[^ ^72^ ^]^ Copyright 2019 The Royal Society. c) Adhesion map (Load parameter vs. elasticity parameter) for elastic sphere based on the Maugis–Dugdale model. In the Hertz zone, adhesion forces are negligible. Reproduced with permission from.^[^ ^67^ ^]^ d) Force‐distance measurement between a diamond tip and Si(111) surface. e) Comparison of the work of adhesion (*w*_adh_) calculated by the pull‐off force method (*w*_adh, PO_) and the integral method (*w*_adh, I_). The dashed vertical line indicates the approximate transition from the JKR to DMT regimes. The grey lines are the best‐fit lines in each regime. Reproduced with permission.^[^ ^81^ ^]^ f) Force at the power 2/3 versus deformation relationship of a cell indented with a colloidal probe. The data reveals two linear regimes of the curve with different Young's modili. The inset shows the force‐deformation curve of the same indentation data. Reproduced with permission.^[^ ^91^ ^]^ Copyright 2008 Springer Nature.

**Figure 4:**
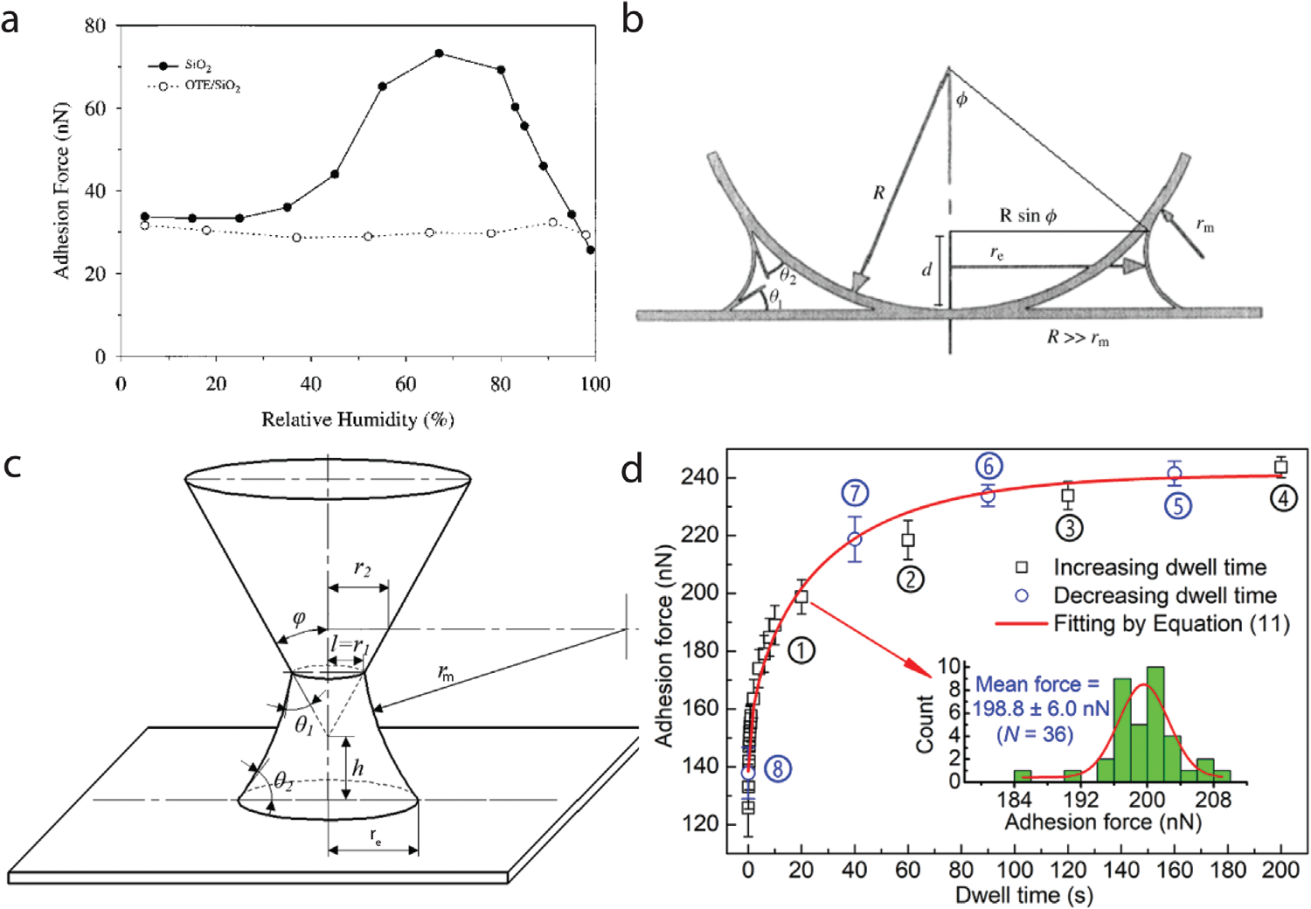
a) The measured adhesion force as a function of the relative humidity between a Si_3_N_4_ tip and a hydrophilic SiO_2_ or hydrophobic with OTE covered SiO_2_ surface. Reproduced with permission.^[^ ^111^ ^]^ Copyright 2000 American Chemical Society. b) The shape of the capillary neck formed between a spherical and a flat surface. Reproduced with permission.^[^ ^101^ ^]^ Copyright 2005 Taylor & Francis. c) Schematic diagram of the meniscus with cone‐plane contact, with *l* the azimuth radius, *h* the gap height, *r*_1_ the radius of the circle formed by the intersection line of the meniscus and conical surface, and ϕ the half cone angle. Reproduced with permission.^[^ ^124^ ^]^ Copyright 2022 American Chemical Society. d) Adhesion force as a function of dwell time (*t*_d_) at 50% RH. The measurement order is represented by the numbers with circles. Each point is an average of 36 measurements when *t*_d_ ⩽ 20 s (4 measurements when *t*_d_ ⩾ 40 s), and the error bar is the standard deviation of each set of points. The inset shows the distribution of data measured with a *t*_d_ = 20 s. Reproduced with permission.^[^ ^129^ ^]^ Copyright 2018 American Chemical Society.

**Figure 5:**
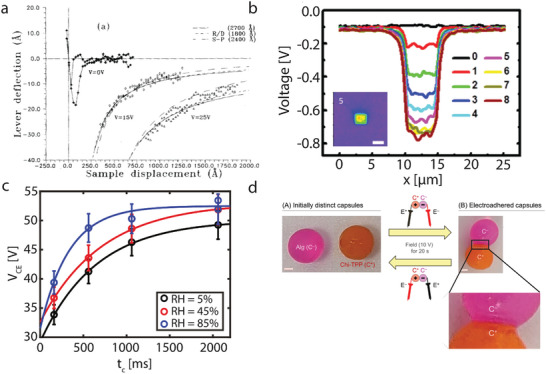
a) The deflection of the lever as a function of the tip‐sample distance without (*V* = 0) and with electrostatic interaction for a tungsten tip and a graphite surface. The measured data is fitted with the macroscopic tip model (solid), the sphere‐plane (SP) model (dashed), and the simple R/D force (dashed‐dotted). Reproduced with permission.^[^ ^154^ ^]^ Copyright 1991 American Institute of Physics. b) Potential profile of the triboelectric charge accumulation on the SiO_2_ surface with increase of the number of repeated rubbing cycles at the same location. Inset: KPFM image after 5 rubbing cycles showing the presence of charge on the rubbed area, scale bar is 5 µm. Reproduced with permission.^[^ ^171^ ^]^ Copyright 2013 American chemical Society. c) The contact electrification voltage vs contact time between a silica colloid in contact with a CF_*x*_ layer for different relative humidities. Reproduced with permission.^[^ ^33^ ^]^ Copyright 2023 American Chemical Society. d) Reversible EA of an anionic Alg capsule to a cationic Chi‐TPP capsule. left: Initial capsules and right: capsules after EA using 10 V DC. Reproduced with permission.^[^ ^182^ ^]^ Copyright 2023 American Chemical Society.

The intermediate regime is described by the Maugis–Dugdale (M–D) theory, which is based on an approximation of the work of adhesion.^[^ ^67^ ^]^ The Maugis–Dugdale theory can be expressed in terms of two non‐dimensional parameters: an elasticity parameter λ related to μ as λ = 1.16μ and a load parameter *P*_L_ defined as *P*_L_ = *F*_App_/*F*_con_ describing the ratio between the applied force and the adhesion force. Using the M–D theory, Johnson and Greenwood constructed an “adhesion map”^[^ ^68^ ^]^ based on the load parameter and the elasticity parameter (**Figure** **3**c). For high load parameters, the adhesion force is negligible, and the Hertz model sufficiently describes the process. Most practical applications fall in the JKR zone of the map, but at the nanoscale many processes operate at values of the elasticity parameter, which pertain to the DMT and M–D zone. Heim et al.,^[^ ^69^ ^]^ corroborated the validity of the DMT theory on a smaller scale by evaluating the pull‐off force between different radii silica spheres, resulting in a clear linear relationship.

The theories above neglect the influence of roughness on the process. Especially on the nanoscale, roughness has a large impact on the contact mechanics force. Contact between rough surfaces will always occur at or near the peaks of the contacting asperities and affect the contact area, i.e., the real contact area will generally be much smaller compared to the apparent contact area. More information can be found in the literature.^[^ ^62^, ^70^, ^71^, ^72^ ^]^

#### 2.2.4 Macroscopic Determination of the Contact Mechanics Force

Johnson et al.^[^ ^64^ ^]^ conducted experiments on the macroscopic scale to test the proposed theories described above. Using rubber and gelatin spheres (spheres with a low elastic modulus to avoid the influence of asperities) and a smooth glass plate (to avoid roughness deviations), they were able to observe the radius of the contact area as a function of the contact force using an optical microscope. Peeling is another method that allows the study of contact mechanics.^[^ ^73^, ^74^ ^]^ The indentation test is the most used contact mechanics measurement on the macroscale.^[^ ^75^ ^]^ A wedge or another tip geometry is pushed into and subsequently pulled out of the sample. During the pulling cycle, the force of the indentor is monitored to determine the adhesion. More advanced setups are, for instance, used for studying the contact mechanics of joints.^[^ ^76^ ^]^ Another approach includes the optical detection of detaching particles from surfaces mounted onto a piezotransducer.^[^ ^77^ ^]^ The surfaces can be oscillated with high frequencies, and the detachment force can be calculated from the particle size and the acceleration due to the oscillation. However, the optical detection limit is determined by the particle size, and no load can be applied to the particle.

#### 2.2.5 Determination of the Contact Mechanics Force at the Nanoscale

On the nanoscale, only two experimental setups are used to study the contact mechanics force. The first is the surface force apparatus (SFA),^[^ ^78^, ^79^ ^]^ which uses two crossed smooth cylinders, mathematically equivalent to a flat surface in contact with a sphere. Although the contact area is in the nanometer range, no other contact geometries can be investigated. The second and more versatile experimental method to investigate the contact mechanics force is AFM. Using AFM, the M–D theory was validated on the nanoscale between a silicon tip and NbSe_2_ sample.^[^ ^80^ ^]^ By changing the tip geometry and material, different areas in the adhesion map (Figure 3c) can be accessed. Moreover, also the nature of the probed surface influences the position in the adhesion map. In Figure 3d, a force‐distance curve is shown of a diamond tip on a Si(111) surface at 1% RH.^[^ ^81^ ^]^ When approaching the tip, the force between the tip and surface gradually becomes more adhesive until the two materials touch (*D* = 0). That only a slight hysteresis was observed between the force on approach and withdrawal is consistent with the presence of only van der Waals or electrostatic forces. The work of adhesion is calculated in two different ways, i) by the pull‐off method and ii) by the integration method. In the pull‐off method, the pull‐off force (the minimum in the force‐distance curve) is linearly related to the work of adhesion *w*_adh, PO_ (see Equation (6)).^[^ ^82^ ^]^ For the second method, the area of the attractive part of the force‐distance curve (negative forces) is determined^[^ ^83^ ^]^ and divided by the contact area to determine the work of adhesion (*w*_adh, I_). In Figure 3e, each data point represents a measure of the work of adhesion every 4 min in the same location. Both work of adhesion parameters commensurately decrease over time which can be ascribed to changes at the interface, e.g., from contact electrification, oxidation or contamination. A clear transition from the DMT to JKR theory is observed. This is only possible because the adhesion forces are conservative forces (van der Waals and electrostatic forces). That is, the amount of energy dissipated during the formation and rupture of the tip‐sample contact was insignificant. However, the moment a water layer comes into play, and the capillary force becomes dominant, the transition between the two regimes vanishes.^[^ ^81^ ^]^ By changing the surface or the tip material/coating, the work of adhesion and thus the contact mechanics force can be determined for different material combinations.^[^ ^84^, ^85^, ^86^ ^]^ This method can also be extended to investigate particle–particle interactions.^[^ ^87^ ^]^

#### 2.2.6 Evaluation of the Experimental Measurement of the Contact Mechanics Force

Biggs and Spinks used the colloidal probe technique to investigate the influence of the load on the adhesion between a polystyrene sphere and a mica surface.^[^ ^88^ ^]^ They found an increase in the adhesion with increasing load or contact time due to plastic deformation of the polystyrene sphere. This result was reproduced on a Si wafer, and using SEM imaging, the deformation of the polystyrene sphere was visualized.^[^ ^89^ ^]^ Similar deformations were found on a PDMS surface after indenting it with a glass sphere.^[^ ^90^ ^]^

In general, both the JKR and DMT theory are often applied to extract the Young's modulus of the sample from the force‐distance curves^[^ ^91^, ^92^, ^93^ ^]^ or to determine the contact area of the tip‐sample contact.^[^ ^94^, ^95^ ^]^ By applying Equation (4) (or the related JKR or DMT equations) to the force‐distance spectroscopy measurements, the Young's modulus can be determined. An example is shown in Figure 3f where the force (*F* ^2/3^) is plotted versus deformation of a colloidal tip on an ovary cell. Using Equation (4), the Young's modulus is extracted from the slope of the line. It can be inferred from Figure 3f that the Young's modulus changed during the measurement as two distinct regimes can be observed. This is often seen for cells, in which first the membrane is indented before the interior of the cell is pushed.^[^ ^96^, ^97^ ^]^ Using a lock‐in amplifier, a spatially resolved map of the Young's modulus can be measured, for instance of small molecular patches.^[^ ^98^ ^]^

Thus, the contact mechanics force plays a huge role in the assembly of nano‐ and microparticles,^[^ ^93^, ^99^ ^]^ and therefore, it is valuable to characterize this force. Solely extracting the contact mechanics force from AFM data remains a tedious task. Especially when other forces are in play, such as the capillary and electrostatic force, the pull‐off force and the work of adhesion are no longer only described by the contact mechanics and van der Waals force, increasing the difficulty to disentangling their influences. However, by performing the experiments at low relative humidities^[^ ^34^ ^]^ or by involving hydrophobic materials,^[^ ^100^ ^]^ these problems can be circumvented, and the influence of the material properties on the contact mechanics force is revealed.^[^ ^93^ ^]^ Therefore, AFM remains the preferred state‐of‐the‐art technique to characterize the contact mechanics force on the nano‐ and microscale.

### 2.3 Capillary Force

#### 2.3.1 Introduction to the Capillary Force

If a water vapor is introduced in the system when two bodies touch, water will adsorb, thus changing the interface energies. At a certain relative vapor pressure, capillary condensation will occur at the point of contact between the two materials, even if one of the materials is hydrophobic,^[^ ^101^, ^102^ ^]^ forming a liquid meniscus between the two surfaces. In practice, it means that the liquid meniscus is formed due to the presence of water in the ambient air. Therefore, in dry assembly, this force can not be neglected on the nano‐ and microscale due to the high surface‐to‐volume ratio.

Various techniques have been used extensively for the analysis of water films on surfaces, such as ellipsometry,^[^ ^103^ ^]^ electron energy loss spectroscopy,^[^ ^104^ ^]^ surface force apparatus,^[^ ^105^ ^]^ and of course AFM.^[^ ^34^, ^106^, ^107^, ^108^ ^]^ Despite of all these techniques, it remains a great hurdle to quantify the interaction strength of the capillary at these small scales. Similar to the situation for the contact mechanics force, the SFA and AFM techniques are the most suitable. Nowadays, the AFM is the most used system to determine the capillary force at these small length scales, due to the geometric degrees of freedom of the system, the controlled environment and the outstanding force resolution.

By performing adhesion measurements under different relative humidity conditions, the effect of the liquid meniscus on the adhesion force is investigated. It has been reported that surface wettability significantly affects adhesion, indicating that the capillary force is often the most dominant force when a hydrophilic body is present.^[^ ^33^, ^34^, ^109^, ^110^, ^111^ ^]^ In the experiments performed by Xiao et al.,^[^ ^111^ ^]^ (**Figure** **4**a), no dependence was found between the maximum adhesion and the relative humidity for a hydrophilic Si_3_N_4_ tip in contact with a hydrophobic surface. However, when the surface was changed to a hydrophilic one, the adhesion was constant up to ≈30% RH. For RH values of 30–70%, a sharp increase in the adhesion was observed, which, reduced for RH > 70%. The adhesion increment can be attributed to the formation of the liquid meniscus (or liquid bridge) at the contact area between the tip and the surface (Figure 4b).

#### 2.3.2 Description of Capillary Forces by the Laplace and Kelvin Equations

From the above, it can already be inferred that understanding the capillary force is essential in assembling particles without solvents. The theory of interparticle forces due to capillary forces and their dependence on relative humidity has been summarized by Israelachvili.^[^ ^37^ ^]^ The smallest radius of curvature of the meniscus (*r*_m_, see **Figure** **5**b) is related to the relative vapor pressure (ρ/ρ_sat_, RH for water) by the Kelvin equation^[^ ^112^ ^]^

(8) $${\left(\frac{1}{r_{m}}+\frac{1}{r_{e}}\right)}^{-1}=r_{k}=\frac{\gamma_{\text{lv}}V}{R_{g}T\log\rho /\rho_{\text{sat}}}$$

where *r*_k_ is the total radius of curvature, *r*_e_ is the contact radius of the liquid bridge, γ_lv_ is the liquid–vapor surface tension, *T* is the temperature, *R*_g_ is the gas constant and *V* is the molar volume of the liquid. The radius of the liquid bridge is described by

(9) $$r_{e}=\sqrt{2Rr_{m}\left(\cos\theta_{1}+\cos\theta_{2}\right)}$$

with θ_1_ and θ_2_ the contact angles of water on the two materials in contact, respectively. For low relative humidity, the Kelvin radius is small, and the capillary condensation first occurs in cracks and pores. In general, *r*_m_ ≪ *r*_c_ holds at which the Laplace pressure is approximated by

(10) $$P=\gamma_{\text{lv}}\left(\frac{1}{r_{m}}+\frac{1}{r_{c}}\right)\approx \frac{\gamma_{\text{lv}}}{r_{m}}$$

and acts on the area of the meniscus of $\pi r_{c}^{2}$, pulling the surface together. For a sphere‐on‐flat geometry (*r*_e_ ≪ *R*, Figure 4b), *r*_e_ disappears from the final expression (and hence the RH dependence) for the Laplace pressure contribution to the adhesion force, given by

(11) $$F_{c}=4\pi R\gamma_{\text{lv}}\cos\theta$$

where θ is the contact angle of the liquid on the two surfaces (assumed identical). When the two bodies are getting separated, the liquid bridge first remains intact. This simple situation is no longer described by Equations ((9), (10), (11)), but its complexity is captured by other formulas.^[^ ^113^ ^]^

#### 2.3.3 Measurements of the Capillary Force

The validity of Equations ((9), (10), (11)) was tested using a SFA.^[^ ^114^, ^115^, ^116^ ^]^ These studies confirmed the Kelvin equation (Equation (8)) and corroborated the increase in adhesion with relative humidity for two hydrophilic mica surfaces. However, the contact areas are relatively large, and with this setup, it is not possible to study particle–particle interactions. Therefore, the AFM is preferred in contemporary studies as one can leverage the advantages of AFM: the contact area is reduced as well as particle adhesion can be studied by attaching particles to cantilevers in the so‐called colloidal probe technique. The measured pull‐off forces and adhesion often fall well below theoretical predictions due to surface roughness, and the comparable size of the contacting radius and the meniscus radius(*R* ∼ *r*_m_ ∼ *r*_c_).^[^ ^117^, ^118^, ^119^ ^]^ It is now commonly accepted that the adhesion magnitude of the liquid bridge depends on a complex interplay of many effects, notably the geometry of surface asperities, meniscus radii, contact angles, and the thickness of adsorbed water films.^[^ ^34^, ^120^ ^]^ Capillary bridge effects only approach their theoretical magnitude when meniscus radii and the thickness of adsorbed water films start exceeding the average asperity size.

#### 2.3.4 Cone‐on‐Flat Description of the Capillary Force

Equation (11) only predicts the maximum magnitude of the adhesion for smooth contacts and is RH independent due to the restriction that the two surfaces must be in contact, i.e., they are physically touching. Moreover, a strong dependence of the adhesion force is often found with respect to the relative humidity (see Figure 4a).^[^ ^34^, ^109^, ^117^, ^121^ ^]^ Therefore, for particle–particle and particle‐surface adhesion, the cone‐on‐flat model is often used. In this model, the surface tension component of the adhesion is included (given by 2π*r*_c_γ_lv_), which becomes dominant at higher RH.^[^ ^122^, ^123^ ^]^ The total capillary force is then given by a capillary pressure term (*F*_p_ determined by the pressure difference inside and outside the meniscus due to the curvature) and a surface tension term (*F*_s_). The capillary force is now written as Ref. [124]

(12) $$F_{c}=F_{p}+F_{s}=\pi \gamma_{\text{lv}}r_{1}\left[2\cos\theta_{1}-\varphi +r_{1}\left(\frac{1}{r_{m}}-\frac{1}{r_{1}}\right)\right]$$

with *r*_1_ is the radius of the intersection line between the meniscus and the cone and φ the half‐cone angle (see Figure 4c).

#### 2.3.5 Disjoining Pressure

Although the cone‐on‐flat model (Equation (12)) describes the adhesion process on the nanoscale better, it still fails to explain the drop in adhesion for high RH values (RH > 80 %). This drop is attributed to the disjoining pressure introduced by Derjaguin.^[^ ^125^ ^]^ The disjoining pressure is the pressure difference between the confined region of two interacting surfaces and surrounding bulk fluid, resulting from the overall surface forces.^[^ ^126^ ^]^ The exact position of the maximum adhesion as a function of relative humidity is determined by the precise tip shape and the roughness of both tip and surface.^[^ ^127^ ^]^

For high relative humidity, the capillary force is changing with time, adding even more complexity to the system.^[^ ^110^, ^124^ ^]^ With time, the adhesion force increases due to the growth dynamics of the liquid bridge. Generally, there are two mechanisms that contribute to the growth of the liquid bridge: i) vapor condensation from the air and ii) the flow of a thin water film toward the liquid bridge.^[^ ^128^, ^129^ ^]^ An example is given in Figure 4d, where the adhesion is plotted as a function of the dwell time (*t*_d_). With the increase of the dwell time, the adhesion force first sharply increases (0–20 s), after which the adhesion slowly saturates (20–200 s). The increase in the adhesion force can be ascribed to an increase in the capillary force (Equation 12). First, the Laplace pressure is much larger because of the small size of a water bridge (first term of Equation (12)). Therefore, the small film of water surrounding the water bridge will flow at a high rate into the contact zone, increasing the capillary force. Due to the increase of the liquid bridge area, the Laplace pressure drops while the disjoining pressure increases because the thickness of the water film decreases due to the flow into the contact zone. This process slows down the increase in adhesion, till the driving pressure gradient vanishes and the water bridge reaches saturation. The process is, therefore, well described with the following model with an initial adhesion force (consisting of the van der Waals, the contact mechanics, and the meniscus force for a small water bridge) and a component dependent on the contact time.^[^ ^129^ ^]^

#### 2.3.6 Evaluation of the Role of the Capillary Force

Especially for hydrophilic systems (hydrophilic particles or surfaces), the capillary force plays a dominant role. Calculating the capillary force strength for particle assembly remains challenging due to the many bodies involved in the process, the different geometries and the various wettabilities. Also, the direction of the movement of the objects has a large influence, as removing a particle from a surface in a humid environment is much more difficult compared to moving the particle along the surface because the liquid bridge remains intact. In addition, as discussed above, the capillary force is dynamic and is highly dependent on the separation distance between the bodies, making it even more difficult to approximate the impact of the capillary force on the assembly. However, under a low RH environment (RH < 5%), the presence of water can be excluded, and therefore the capillary force can be neglected.

### 2.4 Electrostatic Forces

#### 2.4.1 Introduction of the Electrostatic Force

Electrostatic forces are frequently present in particle assembly, in solution and in dry circumstances. While charging of particles in a solution is mainly governed by the solution,^[^ ^130^ ^]^ the main source of charge in dry particle assembly is contact electrification or tribocharging.^[^ ^131^, ^132^, ^133^, ^134^ ^]^. Due to the high surface‐to‐volume ratio, a small amount of charge can induce a large effect on the assembly due to the relatively high strength of the electrostatic force compared to the previously discussed forces.

The electrostatic force (*F*_c_) between two particles is quantified by Coulomb's law

(13) $$F_{e}=-\frac{1}{4\pi \varepsilon_{0}\varepsilon_{r}}\frac{q_{1}q_{2}}{r^{2}}$$

with ϵ_0_ and ϵ_r_ the permittivity of free space and the relative permittivity of the medium in the vicinity of the two particles with charge *q*_1_ and *q*_2_, respectively. The two particles are separated by distance (*d*) from each other.

#### 2.4.2 Measurements of the Electrostatic Force

In order to calculate the electrostatic force, the amount of charge on both bodies needs to be known. Measuring charge on millimeter and sub‐millimeter‐sized particles (or any other type of surface) is usually performed using a Faraday cup and a Faraday cage or any adaptions thereof.^[^ ^132^, ^135^ ^]^ This method is often applied to investigate the charge transfer of aerosol or dust particles to surfaces. For instance, aerosols interact with the walls of dry powder inhalers,^[^ ^136^, ^137^ ^]^ the dust aggregation phase in the formation of planets,^[^ ^138^, ^139^, ^140^, ^141^, ^142^ ^]^ or using electrostatics to remove dust from solar panels in harsh environments.^[^ ^143^, ^144^, ^145^ ^]^ A Faraday cup is usually not suitable for smaller particles due to the high adhesion forces and the small amount of charge present in these systems. In addition, it only measures the total charge in the system and not the exact location where the charge is located.^[^ ^132^ ^]^

In addition to the static approach, also with a dynamic measurement, it is possible to determine the charge of particles (and the particle size distribution) by subjecting the particle to an external electric field and measuring the particle's velocity.^[^ ^146^ ^]^ Electrophoresis can measure the amount of charge and the charge distribution of micron‐sized particles.^[^ ^147^, ^148^ ^]^ Particles are placed in a solution, which flows through a glass capillary over which an electric field is applied. By mapping the trajectory and velocity of the particle, the zeta potential and, thus, the charge distribution on the particle can be determined. The disadvantage is that a solution is necessary to make the particles flow through the capillary, affecting the electrostatic (and all the other) forces.

#### 2.4.3 Kelvin Probe Force Microscopy

Transmission electron microscopy (TEM) and Kelvin probe force microscopy (KPFM) are able to detect charge on the nanoscale. With TEM, the location and the charge density can be determined. However, extensive sample preparation is needed to extract the data.^[^ ^149^ ^]^ KPFM allows the detection of the location and the amount of charge when measuring metals. This technique was initially developed to characterize the local contact potential difference (CPD) between a conductive tip and a metal surface in a closed electrical circuit, thus mapping the work function. More recently, KPFM is also used on dielectrics and insulating particles (and surfaces).^[^ ^150^, ^151^, ^152^ ^]^ Although the location and polarity of the charge can be detected, determining the total amount of charge is extremely challenging. Xu et al., attempted to calculate the number of charges on the semiconductor surfaces, while recently, Waitukaitis and co‐workers reported a method that involved finite‐element method (FEM) simulations in extracting the charge of a flat SiO_2_ layer (3 µm thick) from KPFM measurements.^[^ ^153^ ^]^ As the conversion of KPFM voltage maps into charge remains nontrivial, methods to extract charge from KPFM measurements performed on insulating particles are unprecedented.

#### 2.4.4 Force‐Distance Spectroscopy

On the other hand, Hao et al., studied the long‐range Coulomb forces between an AFM tip and a graphite surface^[^ ^154^ ^]^ using force‐distance spectroscopy (see Figure 5a). When the tip is released from the surface, a force is still interacting on the cantilever, which consists of the van der Waals and electrostatic force. However, remember from Figure 2b that the contribution of the van der Waals force is relatively small, and therefore, this part of the force‐distance curve can be used to fit the electrostatic force.^[^ ^34^, ^58^, ^155^ ^]^ When a voltage is applied to the tip, a larger attractive force is measured between the tip and the surface (Figure 5a), indicating the attractive nature of the electrostatic force.^[^ ^154^ ^]^ First, the sphere‐on‐plane (SP) model was used, which was based on the tip‐surface capacitance and the Coulomb interaction and gives a *R*/*D* dependence.^[^ ^119^, ^156^ ^]^ This model holds for *D* ≪ *R*, but deviates for larger separation distances. Therefore, a more general form for the equation was derived dependent on the geometry of the junction:^[^ ^157^ ^]^

(14) $$F_{e}=\pi \varepsilon_{0}V_{e}^{2}g\left(D\right)$$

with *g*(*D*) the geometrical factor as a function of separation distance *D*. The geometrical factor consists of three components: the apex, cone, and cantilever. All three components contribute in different ways and have different distance dependencies. For other geometries, such as a conical tip or a pyramid shape, the geometry factor is different. Due to the different components, it is regarded as a difficult task to quantify electrostatic forces. Therefore, a useful approximation for all distances is

(15) $$\begin{array}{cc} F_{e} & =\pi \varepsilon_{0}\frac{R^{2}V_{e}^{2}}{D(D+R)} \end{array}$$

(16) $$\begin{array}{cc} F_{e} & =\pi \varepsilon_{0}V_{e}^{2}\left(\frac{R}{D}\right)\mathrm{for}R\gg D \end{array}$$

(17) $$\begin{array}{cc} F_{e} & =\pi \varepsilon_{0}V_{e}^{2}{\left(\frac{R}{D}\right)}^{2}\mathrm{for}R\ll D \end{array}$$

with *V*_e_ the electrostatic potential difference component. Based on this work, besides particle‐surface interactions, also particle–particle interactions are investigated.^[^ ^58^, ^155^ ^]^ Using the electrostatic interaction helped to distinguish between small amphilic positively charged bilayer patches adsorbed on the negatively charged mica surface.^[^ ^158^ ^]^ The difference in the polarity of the charge also showed up in the *F*(*D*)‐curves, where the negatively charged mica surface shows a repelling interaction in contrast to the attractive nature of the electrostatic force on the patches.

#### 2.4.5 Contact Electrification and Triboelectrification

As already mentioned, electrical charges in particle systems mainly originate through contact electrification or triboelectrification. Contact electrification is the process in which two different materials are brought into contact and separated, and charge is transferred from one object to the other. When two materials are rubbed against each other, the process is called triboelectrification. Over the past years, it has been evidently shown that triboelectrification also occurs when identical materials are brought into frictional contact,^[^ ^132^, ^133^, ^159^, ^160^ ^]^ marking its importance in dry assembly and granular matter. Contact electrification is classified into three categories: i) metal‐metal, ii) metal–insulator, and iii) insulator–insulator contacts. Although the charging of metals usually happens unnoticeably because the charge is distributed away from the contact point, charge transfer does occur when two metals are in contact. For metal–metal contacts, in general, the amount of charge transferred is related to CPD, i.e., the difference in the work function of the two materials. Combined with the capacitance between the two bodies, the transferred amount of charge after contact (δ*q*_c_) is approximately given by

(18) $$\delta q_{c}=C_{0}V_{c}$$

where *C*_0_ is the capacitance between the bodies at the critical separation distance and *V*_c_ is the contact potential difference. Experiments so far are in reasonable agreement with the theory.^[^ ^161^, ^162^ ^]^

For metal–insulator and insulator‐insulator contacts, multiple charge transfer mechanisms have been proposed that can play a role. These mechanisms include electron transfer, ion transfer, and material transfer. For some materials, mechanochemical transfer can play a role in which stresses in the material cause bond scissoring and therefore produce radicals, ions, or electrons.^[^ ^160^, ^163^, ^164^, ^165^, ^166^ ^]^ However, given that some of these proposed mechanisms may even occur simultaneously, it becomes a challenge to determine exactly in which direction charge transfer will occur. In this regard, the empirically established triboelectric series guides scientists in modern days in predicting the direction of the charge transfer between bodies of different materials. The series ranks positively charging materials on the top, while materials gaining a more negative polarity can be found at the tail of the series.^[^ ^132^, ^165^ ^]^ Note that the triboelectric series lacks every fundamental basis and deviating results from the prediction are often reported.^[^ ^132^, ^159^ ^]^

Contact electrification or triboelectrification has attracted significant attention recently in light of energy harvesting applications.^[^ ^167^, ^168^, ^169^ ^]^. But, investigating these processes at the micro‐ and nanoscale is challenging due to the limited amount of charge created during the process and the limited time between the charging process and the detection. The charge transfer is, for instance, heavily dependent on the radius of curvature of the particle.^[^ ^170^ ^]^ So far, KPFM is the preferred choice to investigate the charging of two materials in contact at the micro‐ and nanoscale in which the tip of the KPFM acts as one material and the substrate of choice as the second.^[^ ^164^, ^171^, ^172^, ^173^, ^174^ ^]^ An example is shown in Figure 5b, in which the charge accumulation on the SiO_2_ surface is measured after rubbing with a Si or Pt tip. After each cycle, more charge accumulated until the surface was saturated. This relation is explained by the difference in effective work function between the two surfaces^[^ ^171^ ^]^ and matches the results found by Matsusaka et al., for millimeter‐sized particles.^[^ ^175^ ^]^ This method also allows for studying the influence of pressure and contact area on the charging process.^[^ ^176^ ^]^

Recently, the colloid probe technique was explored to investigate the contact electrification between various colloids and surfaces at the nano‐ and microscale.^[^ ^33^ ^]^ The electrostatic component is extracted from the *F*(*D*)‐curves (cf. Figure 2b) using Equation (16). The advantage of this approach is the high time‐resolution, especially between the moment of contact and the moment of the charge detection (<1 s), and the different geometries that can be investigated. It was found that the relative humidity has an important influence on the charging process in line with macroscopic studies.^[^ ^170^, ^177^, ^178^, ^179^ ^]^ The amount of water present on a surface is highly dependent on the curvature of the wetted body, and therefore, the geometry influences the charge transfer process.^[^ ^33^, ^179^ ^]^ The advantage of the colloidal probe technique is the control of the approach, dwell (the time the two bodies are in contact) and retraction time of the process. An example is shown in Figure 5c, in which the contact electrification voltage is measured as a function of the dwell time for varying relative humidity.^[^ ^33^ ^]^ More charge is transferred with longer dwell times, and this process is accelerated within a high relative humidity environment. From these curves, the time constant of charging (τ_d_) can be extracted using refs. [33, 137]

(19) $$Q=CV_{c}\left(1-e^{\frac{-\Delta t_{c}}{\tau_{d}}}\right)$$

with *Q* the contact charge, *C* the electrical capacity of the system, *t*_c_ the contact time and *V*_c_ the potential when in contact. The influence of the dwell time on the electrification process is explained by the velocity of the charge carriers, with a higher dwell time more charge carriers can exchange, and in this specific case, the increase of the electrification voltage is partly facilitated by the dynamic behavior of the liquid bridge (see the similar trend observed for the adhesion force in Figure 4d). When more water is present in the gap, concurrently more charge carriers are present to promote charge exchange. By altering the dwell time, dynamic events can be probed. Adjusting the retraction time of the colloidal probe technique is a trade‐off between a high time resolution and accuracy. A fast retraction time allows us to measure the initial initiated contact electrification charge, but a large number of statistics is necessary to compensate for the inaccuracy in the measurements. The approach and retraction time become more important when non‐Newtonian solutions or visco‐elastic materials are involved due to the shear rate‐dependent viscosity.^[^ ^180^ ^]^ Therefore, one should be careful in choosing the approach/retract velocity and dwell time when utilizing the colloidal probe technique for contact electrification experiments.

Electric fields can also induce adhesion between two different bodies, so‐called electro‐adhesion.^[^ ^181^, ^182^ ^]^ Here, an electric field is applied between a cationic and an anionic material, from which one of the materials is a hydrogel. Using this method, hard and soft (polymeric) materials can be joined together due to an electrochemical reaction that generates chemical bonds between the two electrodes. By reversing the electric field, the adhesion is lifted, and consequently, the two bodies are separated. Using this method, the macroscopic bead chains can be constructed (cf. Figure 5d), but also any other desired geometry.

### 2.5 Evaluation of the Role of the Interaction Forces

Thus, although individual forces can be determined, their interaction strength is affected by each other, posing an immediate challenge to disentangle their influence. For instance, the humidity not only affects the formation of liquid bridges between the particles but also it simultaneously influences the efficiency of contact electrification.^[^ ^33^, ^170^, ^177^, ^178^, ^179^, ^183^ ^]^ Another example, is the effect of the capillary force or liquid bridge on the van der Waals force,^[^ ^184^ ^]^ or the effect of the electrostatic force on the contacts mechanics force and vice versa.^[^ ^175^, ^185^, ^186^, ^187^ ^]^ The combined effect of all these interactions determines the outcome of the assembly of particles.

## 3 Assembly Approaches

**Figure 6:**
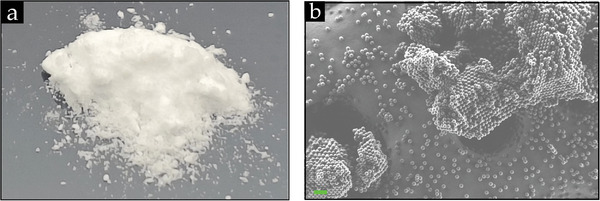
a) A pile of silica powder comprising 10 µm diameter microspheres, and b) a corresponding SEM image of this silica powder. (b) Reproduced with permission.^[^ ^151^ ^]^ Copyright 2022, Royal Society of Chemistry.

The (co‐)existence of the previously discussed surface interaction forces governs the complexity in the dry assembly of ordered particle structures, challenging scientists to exploit various approaches to gain control over these interaction forces. As a self‐explanatory example, **Figure** **6** shows that dry powder, in this case, silica powder, tends to aggregate because of capillary bridges due to their hydrophilic nature. This underscores the first hurdle scientists face as they need accessible building blocks: free individual particles, which they can arrange into ordered structures. It suffices to say that from a fundamental perspective, the dry assembly constitutes a process reminiscent of a phase transition, i.e., from disordered to ordered states. One way to acquire powder containing non‐coherent, discrete particles is by adapting the chemical synthesis of the powder. As Iler reported,^[^ ^188^ ^]^ individual silica particles with a diameter larger than 50 µm could be obtained upon final drying of the acidic solution (pH of ≈2) washed with propyl alcohol. This additional synthesis step prevents the formation of siloxane binding upon mutual contact between particles during drying, which is promoted by the presence of alkali in the solution. However, despite these synthesis adjustments, Iler failed to produce non‐coherent silica particles smaller than 17 µm. The latter can be attributed to the surface interaction forces, mainly the capillary force (see Section 2.3), among the hydrophilic silica particles, becoming more dominant when the particle size decreases. This aggregation in the dry state is also evident from Figure 6b, showcasing 10 µm silica microspheres. Thus, our efforts to generate free individual particles for arranging particle sizes (100 nm to 5 µm) commonly used in colloidal assembly should encompass other approaches.

Therefore, assembly approaches involving long‐range or body forces on the particles are utilized to overcome the short‐range interactions in dry powders. While some scientists solely use a purely mechanical approach, others combine this with electrostatic and acoustic forces. The following reviews a variety of dry assembly techniques.

### 3.1 Rubbing Dry Powder

#### 3.1.1 Rubbing in the Early Days: Fingertips and Oily Rubber Pieces

Manual rubbing dates back to 1972 when Iler reported his pioneering work on rubbing amorphous monolayers comprising silica powder on glass substrates using bare fingertips. It was not popular back in the day due to the poor ordering of particles on a large scale into hexagonal closed‐packed (HCP) ordered crystals. Since then, tremendous progress has been made to advance the manual rubbing method to a level where it is widely favored as a dry assembly approach and employed by scientists to attain crystal structures from dry powder, owing to its ease, simplicity, and rapid assembly time (typically between 5 − 20  s). The introduction of a recent automatic rubbing process may be considered the pinnacle of the rubbing method.^[^ ^189^, ^190^ ^]^

**Figure 7:**
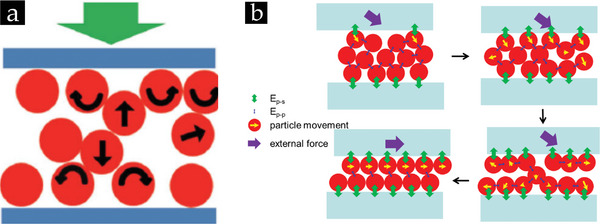
a) Schematic illustration of the induced rolling motion of particles present in the aggregate by applying pressure during rubbing. b) Schematic representation of the separation process of single particles from an aggregate by applying an external shear force that is transferred to each particle in the aggregate while satisfying the condition that the particle‐substrate E_p‐s_ energy should exceed the particle–particle attractive energy E_p‐p_. (a,b) Reproduced with permission.^[^ ^99^ ^]^ Copyright 2014, Wiley‐VCH.

**Figure 8:**
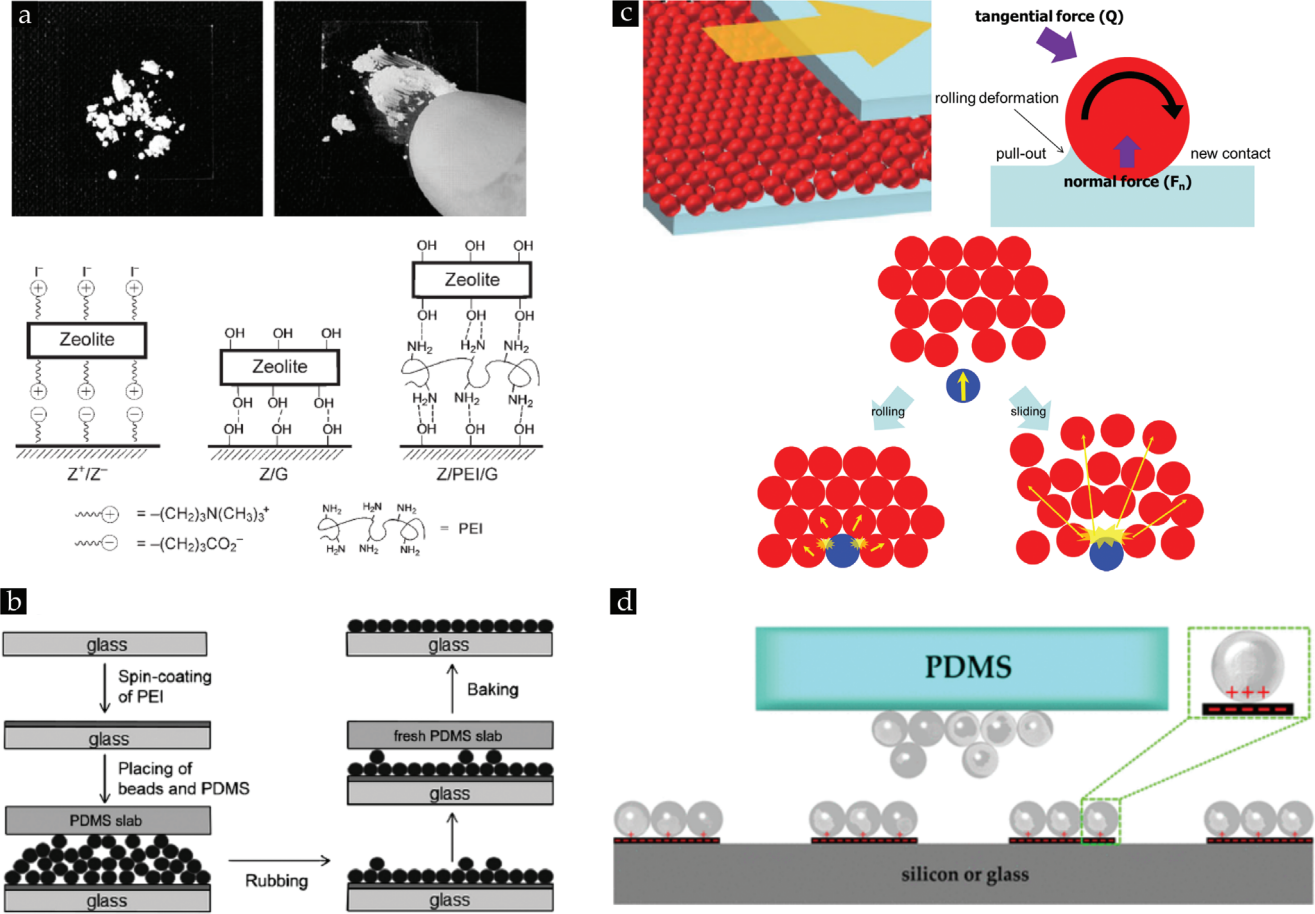
a, top) Rubbing of zeolite powder on substrates using bare finger tips. a, bottom) Schematic illustration of types of effective bonding for the monolayer assembly of zeolite (Z) microcrystals on substrates: ionic bonding between the zeolite‐tethered TMP+ and glass (G)‐tethered Bu^−^, hydrogen bonding between the hydroxyl groups of zeolite and glass, and PEI‐mediated hydrogen bonding between the surface hydroxyl groups of zeolite and glass. Reproduced with permission.^[^ ^192^ ^]^ Copyright 2007, Wiley‐VCH. b) Schematic representation of Rubbing of silica powder using PDMS slabs on substrates covered with a PEI coating that can be removed after the rubbing process. Reproduced with permission.^[^ ^193^ ^]^ Copyright 2012, Wiley‐VCH. c, top) Schematic illustration to attain an ordered monolayer from dry powder that is sandwiched between two PDMS slabs. The formation of a monolayer is mainly driven by a sufficient amount of contact mechanics adhesion due to the soft elastomeric character of PDMS, and (c, bottom) a single crystal is formed when the particles perform a rolling rather than a sliding motion. Reproduced with permission.^[^ ^99^ ^]^ Copyright 2014, Wiley‐VCH. d) Rubbing is performed on a chemically patterned (fluorocarbon) silicon or glass substrate, in which the adhesion is triboelectric charging driven. Reproduced with permission.^[^ ^150^ ^]^ Copyright 2020, American Chemical Society.

During the rubbing process, the coherent powder comprising smaller particles can, for example, be separated into discrete particles by applying a sufficiently strong pressure. With the applied pressure, it is attempted to conceive a transferable momentum to all particles in the aggregate, inducing movement and a continuous rolling motion of particles,^[^ ^99^ ^]^ as illustrated in **Figure** **7**a,b. Next to this, Figure 7b highlights that particle‐substrate interactions must transcend the interparticle forces during the rubbing motion, ensuring that a monolayer of particles is captured on the substrate while the other particle layers move across it.^[^ ^99^ ^]^ To establish this strong adhesion between the particles and underlying substrates for assembling monolayers, different concepts have been explored (cf. **Figure** **8**).

**Figure 9:**
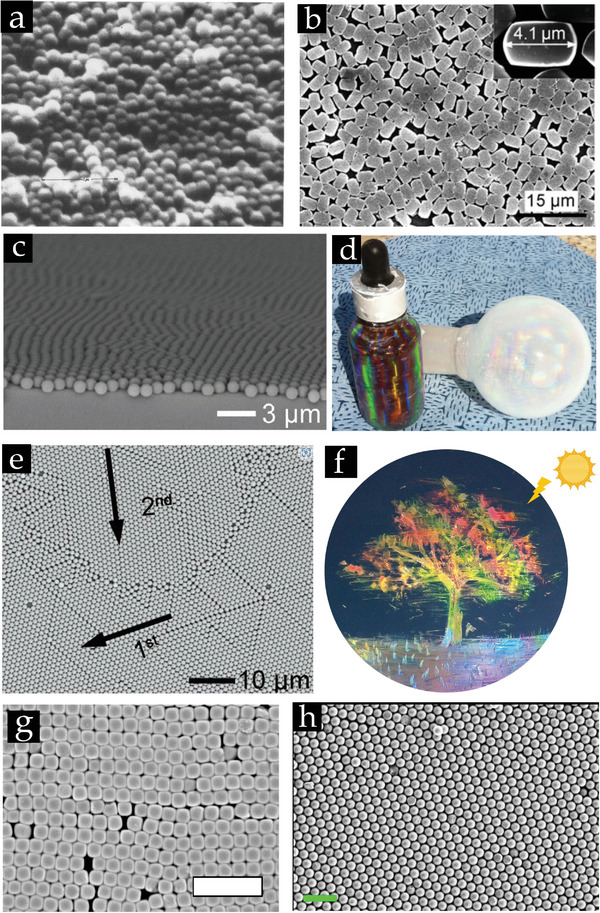
An overview of particle monolayers attained on various surfaces after rubbing dry powder using (a,b) fingertips or (c–h) PDMS surfaces. a) SEM image of a glass substrate carrying a monolayer of 150 µm silica particles with multilayers scattered on top of it. Reproduced with permission.^[^ ^188^ ^]^ Copyright 1972, Elsevier. b) SEM image of a monolayer comprising zeolite crystal on PEI substrates. Reproduced with permission.^[^ ^192^ ^]^ Copyright 2007, Wiley‐VCH. c,d) Single‐crystal monolayers of 1 µm PS attained on (c) flat (SEM image) and (d) curved substrates covered with a PDMS layer. The iridescence color patterns are clearly visible to the naked eye. (c‐d) Reproduced with permission.^[^ ^99^ ^]^ Copyright 2014, Wiley‐VCH. e) SEM image showing a monolayer comprising different crystal orientations attained by changing the rubbing direction using a PDMS pen. f) A macroscale image shows that structural coloration was achieved by adapting the rubbing direction. (e,f) Reproduced with permission.^[^ ^194^ ^]^ Copyright 2015, Springer Nature. g) SEM image of cubic silica particles monolayer on a PDMS substrate attained using automatic rubbing (scale bar: 5 µm). Reproduced with permission.^[^ ^189^ ^]^ Copyright 2021, MDPI. h) SEM image of 3 µm PS beads assembled on an Au‐coated substrate (scale bar: 15 µm). Reproduced with permission.^[^ ^93^ ^]^ Copyright 2021, American Chemical Society.

Starting from rubbing discrete hydrophilic silica particles in a circular motion on glass substrates, Iler observed film formation of the larger particles (>50 µm), albeit amorphous single layers with a few silica particles scattered on top, as shown in **Figure** **9**a. It was postulated that, in this case, the strong adhesion between the silica particles and glass substrates originated from relatively more hydrogen bond formation between the silanol groups at the mutual contact point of the particles and the glass surface compared to the bonds between the scarcely present particles on top. Iler also observed that the pressure applied during rubbing needed to be adapted depending on the particle size to assemble a monolayer. This exhibits characteristics of the contact mechanics force, particularly the DMT model (see Section 2.2), between the silica particles and the glass substrates, which depends on the size of the particle and the applied pressure. A greater contact area at a higher applied pressures increases the adhesion force between the particles and glass substrates. Thus, apart from the proposed chemical mechanism by Iler, the physical concept of the contact mechanics force should have been accounted for in the underlying mechanics for a stronger adhesion between the particles and substrates.

Dimitrov et al.,^[^ ^191^ ^]^ improved Iler's work by utilizing freshly prepared silicon rubber pieces as the rubbing device instead of bare fingertips to assemble amorphous monolayers of silica colloids between 100 − 1000 nm on various surfaces, including glass, leather, and painted cloth. One key finding of this work conducted by the Nagayama group was that the condition of the rubbing devices was pivotal in attaining these amorphous monolayers. In particular, dry rubbing devices failed to assemble, while slightly oily rubber pieces worked best. A second observation was, that their study also revealed that the applied pressure should be adjusted with the particle size, particularly for the larger particles, indicating that the contact mechanics force has a significant contribution. The former observation led the researchers to hypothesize that silicon oligomers were present at the rubbing surfaces and that charging of the system could also explain the established adhesion between particles and different surfaces and, concomitantly, the formation of the monolayers.

In a similar fashion as Iler, Lee et al.,^[^ ^192^ ^]^ rubbed dry powder using fingertips covered in soft latex gloves, as displayed in Figure 8a. They employed zeolite crystal particles. The aggregated particles with sizes ranging between 500 nm and 12 µm were rubbed on uncoated glass substrates and glass substrates covered with a spin‐coated layer of polyethylenimine (PEI). From Figure 9b, it is inferred that an amorphous monolayer of the zeolite crystals was formed on these substrates. As Figure 8a depicts, the authors also used a chemical concept as the underlying mechanism to explain the adhesion of the zeolite powder and (un)coated glass substrates. In particular, Lee et al.^[^ ^192^ ^]^ noticed that the best adhesion and, concomitantly, the best monolayer formation using the rubbing method was obtained on the PEI‐coated substrates, which the authors attributed to the ionic and hydrogen bonding. However, from a physical point of view, the Youngs modulus of the PEI layer is at least two orders of magnitude smaller than that of glass substrates, implying that the contributions from the contact mechanics force are substantial and can not be neglected. The strong adhesive property of PEI was also exploited by Kang et al.,^[^ ^193^ ^]^ to assemble silica colloidal particles between 110–670 nm using the rubbing method performed with a PDMS slab. They obtained a monolayer encompassing HCP‐ordered crystals, albeit with some defects, on the PEI layer that could be removed from the glass substrate using an annealing step performed at 350 °C (cf. Figure 8b). The fact that a sacrificial PEI layer was essential in aiding the assembly of the silica colloids on glass substrates corroborates that sufficient adhesion between particles and substrates is generated by means of surface deformations, i.e., a contact mechanics force. Unfortunately, a remarkable pitfall of this sequential annealing step in the assembly process is that it is incompatible when polymer particles with much lower melting points, e.g., polystyrene or PMMA, are involved.

#### 3.1.2 Advanced Dry Rubbing Techniques on Elastomeric Surfaces

By accounting for the contact mechanics force and conditions of the rubbing device, the Jeong group^[^ ^99^ ^]^ markedly advanced the manual rubbing technique by rubbing dry powder sandwiched between two soft elastomeric substrates (*E* ≈ 3 MPa), mainly PDMS, as illustrated in Figure 8c. These soft elastomeric substrates enable strong contact mechanics forces between the particles and substrate (*F*_p‐s_) as described by the JKR model (see Section 2.2), contributing to the strong adhesion between particles and substrates. Consequently, they succeeded in assembling single crystal structures of silica and polymer colloids of sizes ranging between 110 nm to 5 µm on flat PDMS sheets and concave glass surfaces coated with a thin layer of PDMS, as shown in Figure 9c,d, respectively. This impressive assembly of crystalline monolayers on flat or concave surfaces can be identified from the diffraction patterned colors in Figure 9d. The method's versatility was exemplified by the ability to assemble mm‐sized steel balls into large areas of crystal structures.

To attain these single crystal structures on a large scale, Park et al., postulated a mechanism in which the particles need to roll across the substrate (cf. Figure 8c).^[^ ^99^ ^]^ Moreover, as Figure 8c depicts, sliding particles disturb an already‐formed HCP‐ordered crystal, mimicking a billiard game. Thus, it can be safely concluded that a nucleation point should exist from which the formation of crystal seeds will be promoted, which is in agreement with the earlier observation of Iler.^[^ ^188^ ^]^ They observed a growing monolayer that reflected specific color depending on the particle size from nucleation sites.

For spherical particles experiencing a pressure *P* and shear force *F*_shear_ during the rubbing process, it is known that they will perform a pure steady‐state rolling motion across the substrate when the rolling friction coefficient *μ*_r_ satisfies the following condition:^[^ ^99^ ^]^

(20) $$\mu_{r}\approx \frac{F_{\mathrm{shear}}}{F_{p}-s+P}\approx 1$$

This condition implies that particles should experience sufficient friction, which can be manipulated by applied pressure and shear force during rubbing, as well as the particle‐surface interaction forces *F*_p‐s_ that determine the adhesion between the particles and substrate. The other studies mentioned above also claimed the influence of the applied rubbing pressure on the monolayer assembly. Park et al.,^[^ ^99^ ^]^ meticulously explored the effect of applied rubbing pressure and rubbing speed on attaining a closely packed monolayer. Their findings show strong evidence that the rubbing pressure and the speed play a crucial role in assembling crystal structures. The rubbing pressure is the most sensitive parameter, as the optimum pressure for 170 nm particles was eight times that of 5 µm particles. The latter observation is in agreement with the contact mechanics force as higher pressures and concomitantly larger deformations are necessary to establish sufficient adhesion between the particles and substrates. If too much pressure is applied or the rubbing is performed too fast, the assembled monolayer is damaged due to scratches or dislocations within the crystal structures or even plastic deformations of the soft particles. As noted before, sufficient adhesion of the particles and the soft elastomeric substrates was also essential to capture and assemble a monolayer. However, Park et al.,^[^ ^99^ ^]^ reported that if the adhesion was too strong or if the surface was perceived as “too sticky,” the ordering of the particles was poor, as the movement of the particles on the sticky surfaces was inhibited. Thus, if the adhesion, rubbing pressure, and speed are optimal, the Jeong group could routinely attain monolayers comprising HCP ordered crystals between the soft elastomeric or rubbery substrates.

Inspired by structural colors in nature, the Jeong group extended this quick particle rubbing approach in a follow‐up study.^[^ ^194^ ^]^ Figure 9e shows that the rubbing direction was adapted to tune the crystal orientations within the same monolayer in a controlled manner to generate these structural colors. The crystal orientation combined with the diffraction angle of light determines the color the human eye perceives. Given the reproducibility and rapidness of rubbing powder between two rubbery surfaces, they succeeded in a fine‐art structural color painting by rubbing 1 µm polystyrene beads on a silicon wafer covered with a PDMS layer using a rubber pen, as shown in Figure 9f.

Recently, Napel et al.,^[^ ^189^ ^]^ used the rubbing approach between two PDMS surfaces to assemble spherical colloids and cubic‐like silica particles. The rubbing experiments were performed using an in‐house built device in which a PDMS cylinder moved dry powder back and forth on a PDMS‐coated glass slide. Similar to the work of Park et al.,^[^ ^99^ ^]^ they assembled closely packed spherical silica colloids on the flat PDMS surface. On the other hand, the cubic‐like particles' positional ordering was weaker perpendicular to the rubbing direction, as can be observed from Figure 9g, which the authors contributed to the formation of a sliding phase for these types of particles. The latter can also explain the poor ordering of the zeolite crystals shown in Figure 9b.

#### 3.1.3 Assembly of Particles on Non‐Elastomeric Surfaces

**Figure 10:**
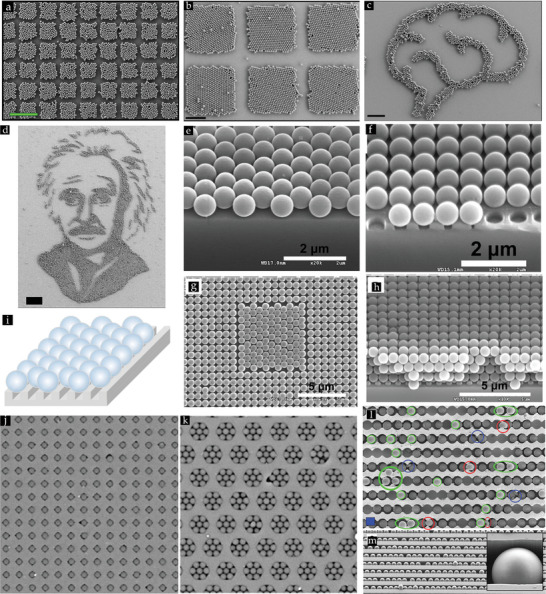
Ordered particle arrays assembled on (a‐d) chemically patterned or (f‐m) physically templated substrates using PDMS rubbing stamps. a) SEM image of 5 µm silica monolayer arrays on fluorocarbon‐patterned glass substrates (scale bar: 100 µm). Reproduced with permission.^[^ ^150^ ^]^ Copyright 2020, American Chemical Society. SEM images of patterned HCP ordered crystals comprising b,c) 10 µm and d) 3 µm PMMA microspheres on fluorocarbon‐patterned 8 µm SiO_2_ substrates (scale bar: 100 µm). (b‐d) Reproduced with permission.^[^ ^93^ ^]^ Copyright 2024, American Chemical Society. e) SEM image of a hexagonal array of 700 nm silica colloids on a flat silicon substrate covered with a positive photoresist layer. f,g) 2D‐ordered arrays and h) 3D‐ordered (five layers) of silica colloids on patterned Si wafer. Reproduced with permission.^[^ ^195^ ^]^ Copyright 2009, American Chemical Society. i) Schematic illustration of spherical PS particles on the line‐and‐space elastomer template. SEM images of 3 µm PS beads assembled in j) circular‐shaped and k) hexagonal‐shaped wells fabricated in PDMS. (i–k) Reproduced with permission.^[^ ^196^ ^]^ Copyright 2016, American Chemical Society. l) SEM image of 10 µm silica microspheres showcasing challenges encountered when rubbing of dry powder is performed for a single time on physically templated silicon substrates: particles forming excess layers or on unwanted positions (green circles), broken particles (red circles), and debris from the PDMS rubbing tool (blue circles). m) SEM image of 5 µm silica beads assembled in silicon‐based channels after three consecutive runs of rubbing. (l,m) Reproduced with permission.^[^ ^197^ ^]^ Copyright 2022, American Chemical Society.

Although the assembly of dry colloidal powder on soft elastomeric substrates (PDMS) presents a promising avenue to rapidly attain HCP‐ordered crystals on a large scale owing to the strong adhesion between particles and PDMS, it also brings some limitations. For instance, it limits broader applicability in, e.g., analytical science or screening where solvents reacting with PDMS deteriorates the sensitivity of assays, or hindering the printing of structured arrays on other substrates due to the stickiness of PDMS.^[^ ^93^, ^190^ ^]^ To overcome these challenges, Jimidar and co‐workers performed an in‐depth study to unravel the underlying physical phenomena occurring during the rubbing process by performing rubbing experiments on nonelastomeric substrates using PDMS rubbing slabs. This study was inspired by earlier work of Jimidar et al.,^[^ ^150^ ^]^ in which they evidently showed that a strong adhesion to capture monolayer arrays of silica microspheres (cf. **Figure** **10**a) can also be established by the tribocharging phenomenon on fluorocarbon‐coated arrays on the wafer depicted in Figure 8d, corroborating the earlier hypothesis, reported more than two decades ago by Dimitrov et al.,^[^ ^191^ ^]^ that charging aids the required adhesion to capture a particle monolayer. The induced electrostatic attraction between the surface and silica particles was supported by performing KPFM measurements.

More recently, Jimidar and co‐workers assembled monolayers comprising HCP ordered crystals of polymer particles (polystyrene and PMMA) with a size ranging between 500 nm to 10 µm on nonelastomeric rigid substrates with an elastic modulus >20 GPa, whereas this was previously only obtained on PDMS (*E* ≈ 3 GPa) or PEI (*E* ≈ 1 GPa) surfaces. The authors reveal that sufficient adhesion can be generated through the tribocharging mechanism and the contact mechanics force. In particular, for stiff substrates on which surface deformations are small, and therefore the contact mechanics force is low, tribocharging is key. In contrast, on substrates where tribocharging can be neglected, the substrate should have a lower Young's modulus to generate sufficient adhesion, as was achieved with 3 µm polystyrene particles on Au‐coated substrates shown in Figure 9h. Furthermore, they revealed that it is essential to have non‐cohesive particles. This claim was supported when they attained HCP‐ordered crystals of silica microspheres by reducing capillary bridge formation between the particles under controlled zero‐humidity glove box conditions, which they failed to produce under standard lab conditions with humidity levels ranging between 40–55%.

Despite the attained monolayers of HCP ordered crystals on the rigid substrates, Sotthewes et al. reported that the assembled monolayers were most stable on the fluorocarbon layer (*E* ≈ 21 GPa) due to the low elastic modulus and its high triboelectrification property.^[^ ^33^ ^]^ On the pristine substrates, the assembled monolayers could be damaged if the applied pressure was too high. Damage also occurred when the rubbing device was not a soft elastomeric substrate. Therefore, they employed chemically patterned substrates to attain patterned HCP‐ordered crystal structures, in which they leveraged the stability of the monolayers on a fluorocarbon layer. As a first step, they assembled a monolayer of crystals across an entire fluorocarbon‐coated SiO_2_ wafer and subsequently removed the particles from the uncoated areas selectively by blowing pressurized nitrogen across the wafer. Consequently, the authors obtained tunable patterns of HCP‐ordered crystals comprising PMMA particles on the fluorocarbon layers in ≈20 s, as showcased in Figure 10b–d, elucidating the stability induced by the fluorocarbon layer.

#### 3.1.4 Rubbing on Physically Templated Surfaces

Apart from employing chemically patterned substrates, the use of physically templated surfaces, i.e., samples covered with periodic holes and wells, is another common approach to attain ordered arrays of colloidal particles. Regarding the latter, Khanh and Yoon rubbed silica colloids using a PDMS stamp on a physically templated silicon substrate covered with a PEI layer, serving as a gluing layer.^[^ ^195^ ^]^ Figure 10f,g shows that the authors could assemble any array on top of the wells covered with PEI, which could be removed by calcining the layer at 500 °C. As previously mentioned, annealing the substrate with particles at these elevated temperatures renders this method inapplicable for polymer particles. To circumvent this, the authors also coated or patterned flat substrates with a positive photoresist, i.e., no physical structures, that could be simply removed by methanol to obtain perfectly ordered 2D arrays of particles (cf. Figure 10e). In the case of photoresist, the rubbing experiments had to be performed gently as this photoresist layer could be damaged due to its weak mechanical stability. In addition, Figure 10h strikingly shows that the well‐structures allowed Khanh and Yoon to attain a multilayer assembly, albeit on a small scale, of 700 nm silica colloidal particles in a square pattern, marking the first time particles were assembled in a 3D crystal orientation (5 layers) using the manual rubbing method.^[^ ^195^ ^]^ As the number of layers increased, the assembly became more challenging due to the instability of the 3D crystal.

Producing physically hard templated surfaces can be costly as it typically requires advanced fabrication techniques developed inside a cleanroom. Another significant drawback of these rigid templated substrates is that they require highly uniform, i.e., monodisperse, spherical particles, since oversized particles could block neighboring particles, restricting the assembly of closely packed arrays or non‐closely packed arrays with minimal small space between neighboring particles. To overcome these challenges with templated rigid substrates, the Jeong group employed physically soft templated substrates in the form of PDMS, as these substrates are more forgiving for particle polydispersity in the perfect assembly of 2D‐ordered crystals due to their flexibility (cf. Figure 10i). This assembly strategy reminisces their earlier work where the dry powder was rubbed between two flat PDMS sheets, replacing one flat substrate with a templated PDMS substrate to attain ordered arrays down to the single particle level (cf. Figure 10j,k).^[^ ^196^ ^]^

As previously mentioned, to prevent contamination, PDMS or other soft elastomeric substrates are undesirable in analytical applications to prevent contamination. Therefore, Verloy et al.,^[^ ^197^ ^]^ employed templated silicon substrates to rub particles within these wells. To exclude annealing or washing steps utilized by Khanh and Yoon,^[^ ^195^ ^]^ Verloy et al., explored a strategy in which assembled particles could be fully sunk and confined within micromachined pockets that were subsequently enclosed with a glass substrate, providing the opportunity to produce a microfluidic device for applications such as liquid chromatography.^[^ ^21^ ^]^ Prior to the rubbing experiments, these hard‐templated silicon substrates were not coated with PEI or other adhesive layers. Consequently, the authors evidently showed the drawbacks of the dry rubbing assembly using PDMS sheets on these templated silicon substrates, as shown in Figure 10l,m. First, pockets remained empty after three consecutive rubbing runs on the substrates. Second, broken silica particles, attributed to the sharp edges of the pockets, could be observed. Third, the sharp edges also caused debris in the PDMS sheets. At last, excess particles are assembled on top of the assembled layers in the pockets. Given these challenges, the authors introduced a wet rubbing assembly technique using a patterned PDMS rubbing tool.^[^ ^197^ ^]^

#### 3.1.5 Evaluation of Rubbing‐Based Approaches

Altogether, the rubbing method is developing into a matured dry assembly field, which started with rubbing using bare fingertips, and now automatic rubbing setups^[^ ^189^, ^190^ ^]^ using PDMS rubbing stamps are being explored. The examples demonstrate that this rubbing approach is able to overcome the strong cohesive interactions while ensuring that sufficient adhesion is generated between the captured colloids and the underlying substrate. A variety of concepts have been reported to promote the adhesion between the particles and substrates, such as the use of elastomeric substrates or substrates that can be tribocharged during rubbing. Different bead sizes, from mm‐sized steel balls to 170 nm PS colloids, have been assembled in <20 s on wafer‐scale elastomeric substrates, elucidating the broad applicability of the simple and rapid rubbing method to attain ordered monolayer or ordered arrays on flat or physically‐templated elastomeric substrates respectively. This example also highlights the advantage of dry assembly over wet assembly. Furthermore, to ensure broader applicability in the (bio‐)analytics domain, fluorocarbon‐coated substrates have been utilized to attain ordered HCP crystal structures. One of the main drawbacks is that an excessive amount of dry powder is needed to assemble the ordered structures, while physically templated substrates of robust materials, e.g., silicon, deteriorate the efficacy of the rubbing method, limiting applications in the microfluidics analytics domain.

### 3.2 Shaken (“Not Stirred”) Particles

#### 3.2.1 Macroscopic Agitation Experiments

Another mechanical approach used to assemble ordered particle structures is the concept of shaking or agitation of a particle ensemble. The supply of mechanical energy in shaken granular media has been utilized to understand the crystallization and phase transformations of grains, with the dedication of scientists from the granular community.^[^ ^198^, ^199^, ^200^ ^]^ Therefore, an infinity of reports exists on attaining structures comprising beads of the same material in an agitating system.

**Figure 11:**
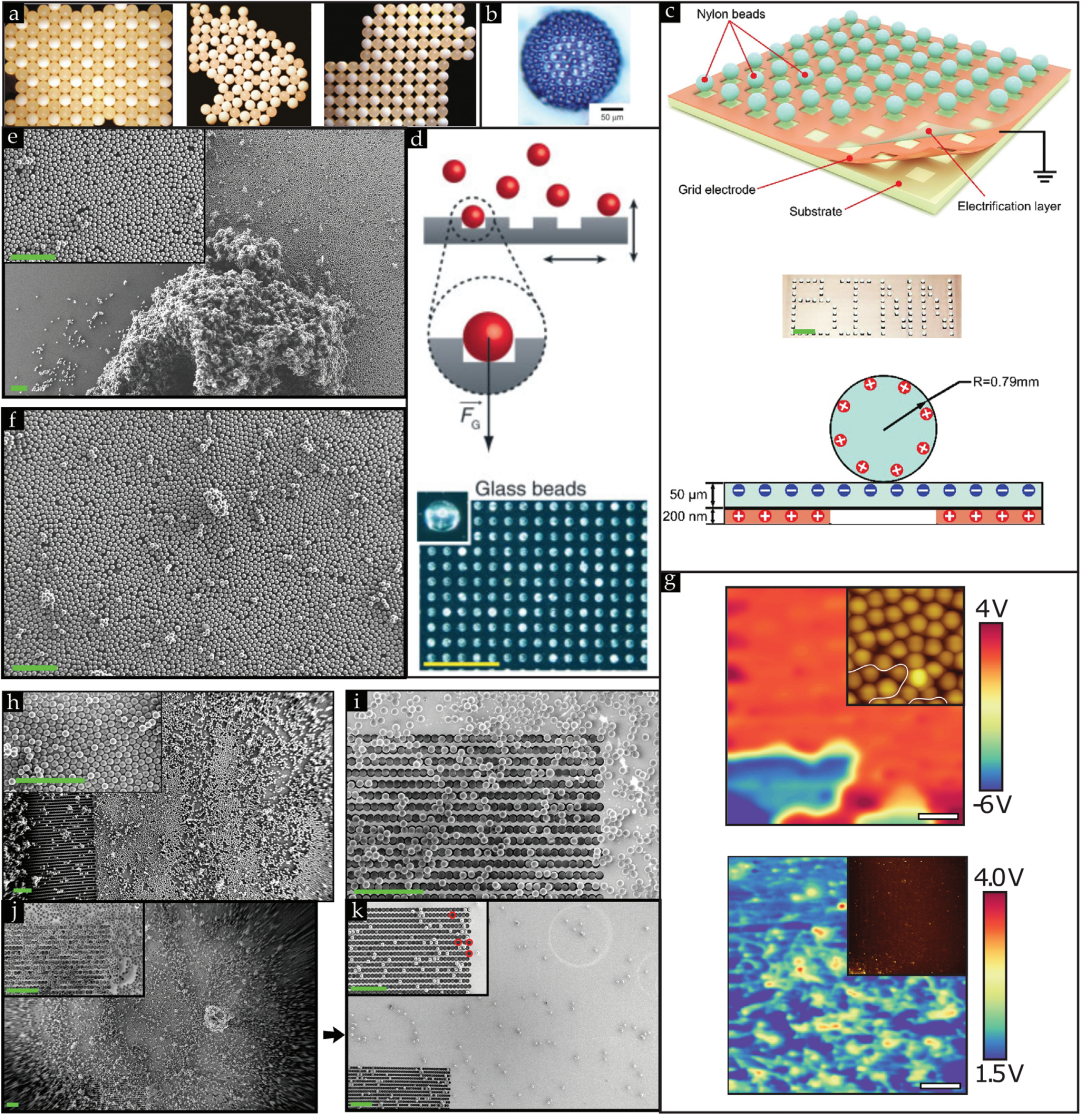
a) Electrostatic assembly of agitated macroscopic crystals comprising a binary mixture of polymer beads on Au substrates. Reproduced with permission.^[^ ^201^ ^]^ Copyright 2003, Springer Nature. b) Electrostatic assembly of a binary crystal comprising cubic polymer objects agitated on an aluminum dish. Reproduced with permission.^[^ ^202^ ^]^ Copyright 2012, Royal Society of Chemistry. c) Schematic illustration of the electrostatic templated self‐assembly of macro‐sized spheres (scale bar: 10 mm). Reproduced with permission.^[^ ^204^ ^]^ Copyright 2018, American Chemical Society. d) Assembly of 100 µm glass beads on physically templated substrates using the agitation concept (scale bar: 1 mm). Reproduced with permission.^[^ ^205^ ^]^ Copyright 2005, Wiley‐VCH. SEM images of agitated 10 µm e) silica and f) PS dry powder on fluorocarbon‐coated silicon substrates (scale bar: 100 µm). KPFM measurements were performed after horizontal agitation on g, top) silica microspheres and (g, bottom) fluorocarbon‐coated silicon substrate. Insets represent the corresponding topography scans (scale bar: 5 µm). (e–g) Reproduced with permission.^[^ ^151^ ^]^ Copyright 2022, Royal Society of Chemistry. 10 µm h) silica and i) PS particles agitated on microstructured silicon substrates (scale bar: 100 µm). 10 µm PS beads agitated on a microstructured silicon substrate j) before and k) after removing the excess particles from the silicon substrate using a PSPMA brush. Red circles represent the undesired capture of particles from the grooves (scale bar: 100 µm). (h–l) Reproduced with permission.^[^ ^206^ ^]^ Copyright 2020, Ignaas Jimidar.

Instead of using homogeneous systems, the Whitesides' group leveraged the tribocharging concept to explore the assembly of binary granular crystals by agitating two distinct millimeter‐sized polymer grains inside a container. Due to the mechanical energy supplied to the system, the grains start moving across the substrates, promoting collision events among the constituents. Consequently, charge exchange occurs between the particles and the particles and container. By cleverly selecting the materials of all these three entities in a series of studies, the Whitesides' group succeeded in assembling 2D granular lattices with square, pentagonal, or hexagonal symmetry comprising spheres or cubes (cf. **Figure** **11**a). The type of assembled symmetry hinges on the ratio of distinct particles, the kinetic energy in the system, and the ability of both types of grains to attain opposing polarities, inducing electrostatic attraction among the grains that outweighs any interaction between these constituents and the container.^[^ ^201^, ^202^ ^]^

This agitation‐driven tribocharging approach, specifically the concept of mobile ion transfer between bodies in contact, to attain 3D ordered microstructures was explored by McCarty et al.,^[^ ^203^ ^]^ The 3D ordered structures displayed in Figure 11b were assembled after large polystyrene (200 µm) and an excessive amount of smaller polystyrene (5 µm) beads, both with distinct functional groups to unlock the chemically directed tribocharging mechanism (see Section 2.4), were shaken in an aluminum dish. When the large and small polystyrene microspheres made contact, they acquired opposing charges, inducing an electrostatic attraction that covered the large beads with the smaller ones. The sharp reader may have noticed this is a remarkable result, as one would expect a Coulombic repulsion among the small spheres with the same polarity sign. However, it is plausible that the electrostatic attraction between the large and small beads surpassed the repulsion among the smaller beads. On the other hand, the stability of these 3D‐ordered structures could be underpinned by attractive induced polarization forces among the smaller spheres.^[^ ^132^ ^]^ Notably, the authors failed to assemble these stable 3D‐ordered microstructures when the dish was covered with an insulator layer, as the small polystyrene beads would stick to it due to the tribocharging phenomenon and the contact mechanics force to some extent.

Wang et al., also exploited the tribocharging concept to direct the assembly of shaken macroscopic nylon beads into ordered, albeit non‐closely packed, arrays.^[^ ^204^ ^]^ As a first step in their assembly approach, they precharged the nylon beads by shaking them in a Teflon container while their substrate carrying a Teflon coating on top of a templated copper electrode was rubbed with an aluminum foil. Consequently, the nylon beads acquired a positive polarity, whereas the substrate's Teflon coating was negatively charged. The latter induced positive charges in the grounded copper electrode, as illustrated in Figure 11c, screening the negative charge at specific areas on the Teflon coating. By gently shaking the charged nylon beads across the substrate, the beads were trapped in spots where the copper electrode was missing underneath the Teflon coating. Figure 11c shows that the approach allowed Wang et al., to obtain arbitrary, predetermined arrays of the millimeter nylon beds.^[^ ^204^ ^]^ This assembly approach reminisces the photocopying technique. Note that due to gravity, a few of the excess particles rolled from the substrate when slightly tilted.

#### 3.2.2 Nano‐ and Microscale Particle Assembly Using Agitation

Moving away from the tribocharging concept, Kraus et al.,^[^ ^205^ ^]^ also employed the agitation approach to assemble 100 µm glass beads on microstructured surfaces or physically templated substrate by leveraging gravity. As Figure 11d shows, the glass beads were shaken horizontally and vertically, and due to gravity, the beads could be captured on the microstructured surfaces.

The aforementioned achievements may suggest that agitation can easily be adapted to attain similar structures with (sub‐)micro‐ and nanoparticles. However, Jimidar et al.,^[^ ^151^ ^]^ observed precisely the opposite when agitating monodisperse silica or polystyrene powder comprising spheres with diameters ranging between 3 − 10 µm on bare and fluorocarbon‐coated silicon substrates in the horizontal direction. One key observation in this study was that a significant amount of energy was required to mobilize the particles, particularly in the case of the strongly aggregated silica powder (cf. Figure 6a). The fact that the mobilization of polystyrene particles was less challenging can be ascribed to the weaker cohesive interactions among polystyrene particles due to their hydrophobic nature. In contrast to preceding observations with larger beads, it can be inferred from Figure 11e,f that amorphous monolayers were attained, i.e., HCP‐ordered crystal structures were not predominately present. These results indicate that the particles' kinetic energy was not commensurate with the adhesion and frictional forces for these particle sizes at the microscale, i.e., particles were stuck on the substrates, implying that higher frequencies and amplitudes were plausibly required to achieve ordered crystal structures. Furthermore, the formation of these monolayers was promoted on the fluorocarbon‐coated surfaces, which was ascribed to the tribocharging phenomenon. The latter was corroborated by state‐of‐the‐art KPFM measurements, as showcased in Figure 11g, showing that evidently, the fluorocarbon‐coated substrate acquired a more negative polarity locally where the particles moved across the surface, highlighting the charge transfer between the moving microspheres and substrate. Consequently, the adhesion between the particles and substrate is enhanced.

It was also attempted to assemble the silica and polystyrene particles on the microstructured silicon substrates shown in Figure 10l. In contrast to the work of Kraus et al.,^[^ ^205^ ^]^ using 100 µm glass beads (cf. Figure 11d), it can be observed from Figure 11h that the 10 µm silica microspheres remained stuck, in monolayers and aggregates, on the silicon substrate due to the strong adhesion between the hydrophilic silica particles and silicon surface, impeding the assembly of the silica particles within the micro‐pockets.^[^ ^206^ ^]^ This corroborates the challenge that the adhesion and even cohesion forces in this ultrafine silica powder are so strong that it is challenging to transcend these interactions using the agitating approach.

On the other hand, as the polystyrene microspheres are less cohesive and weakly interact with the silicon substrate, they could be assembled within the micro‐pockets, with a few empty spots and excess particles remaining on the microstructured substrate, as shown in Figure 11i. However, Jimidar reported that excessive polystyrene powder was needed to occupy 99% of the micro‐pockets on these microstructured silicon surfaces, covering the microstructures with excess particles. To remove these excess particles, Jimidar employed a polymer brush (poly(3‐sulfopropyl methacrylate (PSPMA)) anchored on a silicon surface to capture these excess particles from the surface.^[^ ^206^, ^207^ ^]^ As these polymer brushes could be easily rinsed, the same brush could be pressed again to remove the remaining excess particles.^[^ ^208^ ^]^ The polymer brush exhibited a higher efficacy in removing the particles from the silicon surface compared to particles stuck on top of the microstructured area, while also capturing particles from the pockets (cf. Figure 11j,k). The latter is an undesired effect, highlighting that further research is required to tune the adhesive interactions between the polymer brush and the particle, targeting solely particles on top of the substrate or another particle.

#### 3.2.3 Evaluation of Agitated Particles

The examples above ascertain the dominating effect of surface interaction forces as we move from the macroscale to the microscale to assemble building blocks using the agitating concept. The agitation concept can be employed to assemble particles into monolayers or ordered arrays on pre‐structured surfaces experiencing weakly cohesive and adhesive interactions, albeit with an excessive amount of particles; it also poses a laborious challenge to remove the excess particles. Therefore, a clear limitation for assembling colloids, particularly hydrophilic particles, of 10 µm and smaller using the agitation concept is observed. In the future, this may be solved by utilizing surface acoustic waves devices^[^ ^201^ ^]^ to overcome the strong interaction forces presents among smaller colloidal aggregates.

### 3.3 Electric Field‐Assisted Assembly

Moving away from pure mechanical processes such as rubbing and agitation of particles, we shed light on methods revolving around the application of an electric field. In the preceding sections, we already elaborated that electrostatics can be leveraged in rubbing and shaking experiments to direct the assembly of structures on demand, particularly when templated substrates are involved. As the electrostatic force is effective over a long‐range, dry powder can become responsive by applying an external electric field.

**Figure 12:**
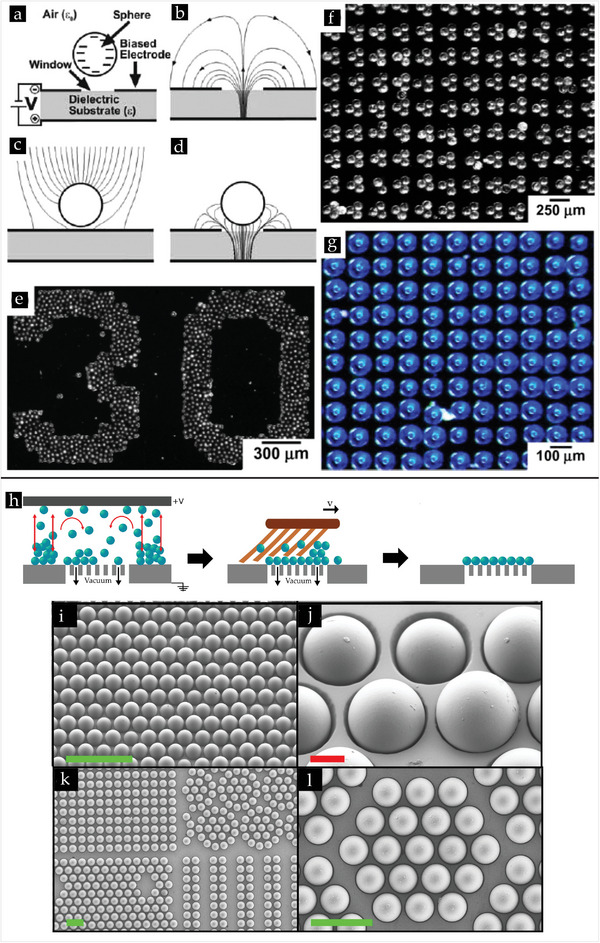
a–d) Schematic representation of the electric field assembly of charged spheres on templated electrodes, and the effect of the field lines in the presence of a sphere. Optical microscope images of e) 40 µm diameter copper shot over large windows (Arabic numerals), f) 100 µm diameter glass spheres assembled over triangular windows, and g) a lattice of blue‐dyed polystyrene spheres (100 µm diameter) dyed with 1,4‐bis(pentylamino)anthraquinone) self‐assembled over PS windows. (a–g) Reproduced with permission.^[^ ^211^ ^]^ Copyright 2008, IEEE. h) Schematic illustration of an electrostatic cell in which microspheres are levitated and subsequently attracted on a microstructured device connected to a vacuum pump. Afterward, excess particles are removed by a brush, and an ordered arbitrary array of microspheres is attained. The corresponding SEM images of i–j) 5 µm silica microspheres, and 10 µm k) PMMA and l) PS particles assembled on microstructured silicon substrates (scale bar: green: 20 µm; red: 2 µm). (h–l) Reproduced with permission.^[^ ^212^ ^]^ Copyright 2022, Elsevier.

In sequential studies,^[^ ^209^, ^210^, ^211^ ^]^ the Whiteside's group fabricated a dielectric substrate with a patterned conductive electrode, e.g., gold, with a thickness of ≈50 nm, as illustrated in **Figure** **12**a–d. After adding a few layers of dry particles on these substrates, a DC‐bias of 10 − 20 kV was applied to trap the beads on the uncoated zone on the substrate selectively. Consequently, the beads were charged with the same polarity as the patterned biased electrode, as the schematic depicts in Figure 12a–d. The application of these high potentials (10 − 20 kV) to induce strong electrostatic forces on the particles was twofold: the applied voltage should ensure that particles were attracted on the windows, i.e., uncoated zones, while particles residing on the conductive electrode needed to be repelled from the electrode. The latter implies that the electrostatic force should surpass the weight of the particles, whereas for particles with a diameter of 10 µm or smaller, adhesion forces dominate, leaving aggregated particles on the entire surface. Thus, it is elucidated that gravity counteracts the assembly process for large spheres, while adhesion forces impede the assembly of smaller particles. The local spatial attraction and the collective ejection of particles from the conductive electrodes driven by electrostatics paved the way to assembled ordered 2D arrays on any predetermined lattice, as displayed in Figure 12e,g, of conductive, semiconductive, and dielectric particles with diameters ranging between 20 − 750 µm. The few remaining excess particles could be removed from the conductive electrode by gently agitating the substrate while applying the DC voltage to fix the position of the trapped particles on the uncoated zones. This entire assembly process spanned a time of 2 − 5 s.

To overcome the limitations of small aggregated dry powder (particle size <10 µm), Van Geite et al.,^[^ ^212^ ^]^ proposed an ingenious approach in which they combine the application of an electric field and a vacuum‐driven force to direct the assembly of monodisperse microspheres between 5 − 10 µm on physically templated microdevices. As Figure 12h illustrates, dry powder was utilized in an electrostatic cell, which was subsequently fluidized by applying strong electric fields (1.2 ⩽ *E* ⩽ 3 MVm^−1^) that could transcend the substantial cohesive interactions among the aggregated particles. Consequently, the particles were ejected from the bottom toward the top electrode with great momentum, where any smaller aggregates would collide and be disrupted into single microspheres, as was previously reported by Jimidar et al.^[^ ^152^ ^]^ Particularly, it was shown that if the top electrode is less adhesive, the single microspheres would bounce from the top electrode, which promoted a particle cloud between the electrodes. To leverage the availability of a single microspheres for ordered assembly purposes, Van Geite et al.,^[^ ^212^ ^]^ designed a bottom silicon electrode comprising microstructured through‐holes, i.e., a silicon membrane, to capture the microspheres by application of a sufficiently strong vacuum pressure (Δ*p* ⩾ 500 mbar). Although many of the particles were positioned on the holes, a few excess particles inevitably remained on the assembled arrays, which could successfully be removed by including a brushing step while maintaining the vacuum force. As a consequence, it was shown that any predetermined lattice of silica, polystyrene, and PMMA microspheres could be assembled (cf. Figure 12i–l within a time frame of 6 − 8 s, including the required brushing step. The authors noticed that electric fields of greater strengths were mainly needed to eject the particles from the bottom electrode in the case of the polymer particles, as their dielectric constant, and concomitantly, their polarizability and induced electrostatic force is lower compared to silica powder.^[^ ^152^, ^212^ ^]^ As will be addressed further in Section 4, the authors could also transfer these particles to other soft elastomeric substrates.

Collectively, these studies demonstrate that the electric field is able to induce the movement of (individual) dry powder particles and attain ordered structures. A significant drawback of this technique is fabricating physically templated surfaces, which can be costly and time‐consuming. However, given that substrates are recyclable, the fast assembly process and precision positioning of particles outweigh the challenges in fabricating these surfaces. On the other hand, it is expected that the assembly of (sub‐)micrometer particles using an electric field may be more challenging for the Coulomb force to overcome the stronger cohesive interactions between the particles. Furthermore, the fabrication of pre‐structured surfaces for colloidal particles demands more sophisticated equipment, posing a limitation for broad applicability in terms of costs and labor intensity.

### 3.4 Other Approaches

**Figure 13:**
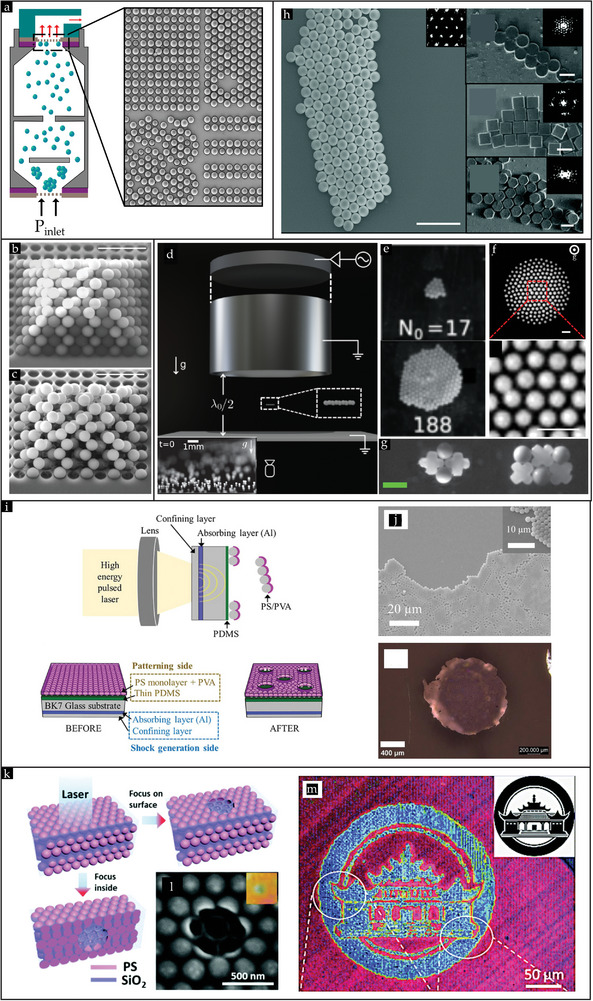
a) Schematic illustration of powder fluidization using *P*_inlet_ to access single microspheres which are attracted to microstructured devices to assemble 10 µm silica powder particles. Reproduced with permission.^[^ ^213^ ^]^ Copyright 2023, Elsevier. b) A mBCC crystal comprising 165 latex and 177 silica microspheres with a diameter of 1.18 µm. c) Same sample as (b) after plasma etching resulting in a diamond lattice (scale bar in b,c: 5 µm). Reproduced with permission.^[^ ^214^ ^]^ Copyright 2002, Wiley‐VCH. d)Schematics of a single‐axis transducer consisting of a piezoelectric driving element coupled to an aluminum horn to generate ultrasonic standing waves in the gap (adjusted to half the resonant sound wavelength (λ_0_ between the bottom of the horn and a reflecting, optically transparent surface. Particles are levitated at the pressure nodal plane in the middle of the gap and imaged with a high‐speed video camera from below. e) Self‐ assembly of granular rafts viewed from below comprising (top) 17 and (bottom) 188 particles. Reproduced with permission.^[^ ^216^ ^]^ Copyright 2022, American Physical Society. f) Image of a levitated particle raft composed of beads of 40 µm (scale bar: 100 µm). (d,f) Reproduced with permission.^[^ ^218^ ^]^ Copyright 2023, Proceedings of the National Academy of Sciences. g) Self‐assembly of lock and key granular clusters composed of spheres and crosses (scale bar: 1 mm). Reproduced with permission.^[^ ^217^ ^]^ Copyright 2023, American Physical Society. h) Representative SEM images of acoustically assembled colloidal crystals after drying (scale bar: 50 µm). Reproduced with permission.^[^ ^220^ ^]^ Copyright 2016, Royal Society of Chemistry. i, top) Schematic illustrations of laser‐induced shockwave spallation technique and specimen structure to generate 2D patterns of PS monolayers, and (i, bottom) illustrations of the sample structure before and after the spallation process. j, top) SEM image indicating the clear grain boundaries, which supports the removal of the PVA layer after the washing. (j, bottom) Optical microscope images showing the spallation of the sample. (i,j) Reproduced with permission.^[^ ^221^ ^]^ Copyright 2024, Wiley‐VCH. k) Fabrication schemes of surface patterns and defects in PS/SiO_2_ CPC films using laser irradiation. l) SEM image of the surface pattern formed on the PS/SiO_2_ CPC films after the laser irradiation (1.5 mW; 60 s). Inset is the bright field optical image of the irradiated spot. m) Optical images of CPC micropatterns (logo) via laser direct writing. (k–m) Reproduced with permission.^[^ ^222^ ^]^ Copyright 2019, Royal Society of Chemistry.

In a follow‐up study, Van Geite et al.,^[^ ^213^ ^]^ explored a different approach to fluidizing dry powder into single particles. Instead of applying an electric field in their electrostatic cell setup, they employed an enclosed chamber where dry powder was shot against an impact plate using an overpressure or a so‐called “pressure shock.” Combined with the vacuum‐assisted assembly method, they attracted particles at the top of the chamber onto the microstructured silicon device to attain similar assemblies as in their previous electrostatic cell study within <8 s (cf. **Figure** **13**a). In contrast to the electric field approach depicted in Figure 12h, the yield of using the“pressure shock” method to make perfect monolayers was lower due to the inability to overcome the particle–particle interactions (due to the contact mechanics and capillary force) and a large majority of particles adhered to the walls of the enclosed chamber. The latter can be ascribed to the triboelectric charging phenomenon, as discussed in Section 2.4. Moreover, it should be remarked that this fluidization approach will restrict the assembly of smaller particles (<5 µm), as it is a hurdle to overcome the cohesive interaction among the aggregated powder particles, whereas the electric field approach elaborated in Section 3.3 is more promising in this regard.

When building structures in our daily lives, we individually pick each object, e.g., marbles or bricks, and place them next to or on each other to realize our desired structure. This macroscopic concept was literally translated by designing a nanorobotic pick‐and‐place setup to assemble non‐close packed 3D‐ordered crystals.^[^ ^214^ ^]^ Silica and polystyrene microspheres with a diameter of 1.2 µm were precisely positioned to designated spots on a predetermined lattice of a microstructured silicon surface using a nanorobot connected to an SEM. The templated silicon surface ensured that the first layer of particles could serve as a robust anchoring layer for the additional layers on top, as shown in Figure 13b. To attain an open 3D structure of silica particles, the PS particles were eradicated by means of oxygen plasma etching (cf. Figure 13c). While this innovative approach is undoubtedly appealing, it is time‐consuming and concurrently not practical for large‐scale applications, as the precise positioning of each particle spanned ≈7 min, such that it is not expected that this approach will be generally adopted, unless one proposes a faster way for positioning particles..

Up to this point, the elaborately discussed dry assembly approaches pertain mainly to the mechanical, electrostatic‐driven, or a combination of both categories. However, we should also shed light on substantial endeavors from the Jaeger group on the acoustic assembly of levitated beads.^[^ ^215^, ^216^, ^217^, ^218^ ^]^ Figure 13d illustrates the induced acoustic trap between an ultrasonic transducer and a reflective surface to assemble levitated polyethylene particles with diameters ranging between 20 − 850 µm. The applied standing sound wave generated a strong sound pressure to levitate the particles at the bottom (cf. inset Figure 13d), which subsequently collided to finally self‐assemble into so‐called “granular rafts” (cf. Figure 13e). The self‐assembled ordered monolayer structures are stabilized by an attractive force that can be attributed to sound scattering among the beads. Moreover, repulsive interactions among the constituents are also prevalent, which arise from viscous fluid interactions, in this case from the air. These repulsive interactions are particularly dominant for small particles, allowing Wu et al., to balance the acoustic scattering and viscous interactions to attain assembled open structures solely for smaller particles (≈40 µm), as shown in Figure 13f.^[^ ^218^ ^]^ In addition, it is striking to observe from Figure 13g that the Jaeger group demonstrated shape‐dependent acoustic levitation to generate specific lock‐and‐key bonds between millimeter‐scale particles.^[^ ^217^ ^]^

While the above is valid for relatively large particles (≈40 µm), single‐grain levitation, and concomitant assembly at the microscopic and nanoscale are challenging as particles aggregate due to the strong cohesive interactions. Although this review's scope is to shed light on assembly approaches without using solvents, we want to emphasize that acoustic assembly is employed to assemble ordered structures from colloidal particles in liquid suspensions.^[^ ^219^, ^220^ ^]^ Owen et al., succeeded in assembling spherical and shape‐anisotropic colloids at the pressure nodes inside the acoustic cell, as showcased in Figure 13h.

Next, laser‐induced techniques have also been explored to attain patterned arrays of 2D colloidal crystals after first assembling a close‐packed monolayer comprising 2 µm polystyrene beads on a thin PDMS layer using the rubbing technique described in Section 3.1. Subsequently, Nguyen et al. covered the monolayer with a thin layer of poly(vinyl alcohol) (PVA). Utilizing a high‐energy density laser generated laser‐induced shockwaves, removing the beads and PVA layer on selective areas from the assembled monolayer (cf. Figure 13i).^[^ ^221^ ^]^ The addition of PVA was a necessity as it promoted the complete removal of the entire solid particle, preventing fragmentation and reattachment of the polystyrene beads. The pattern sizes were controlled by tuning the laser density and including shadow masks with different hole sizes to block the laser beam partially. Figure 13j shows that this laser‐induced shock wave spallation approach is still in its infancy, as millimeter‐sized circular patterns could be achieved but with poor resolution (on the order of a few micrometers).

On the other hand, Wang and Ding utilized the laser direct writing technique to create micropatterns within 3D colloidal photonic crystals that are attained using the wet convective assembly of 240 nm polystyrene colloids.^[^ ^222^ ^]^ Subsequently, a silica sol‐gel was spin‐coated, serving as an inverse opal skeleton structure to stabilize the crystal when removing the polystyrene particles. By leveraging the selective photodegradation of polystyrene, Figure 13k,l depicts that patterns could be created on the surface or within the photonic crystal, depending on the focus area of the laser. As Figure 13m showcases, micropatterned high‐resolution images (10 000 DPI) were generated by applying different laser powers and irradiation times when the photonic crystal was translated on a stage in a controlled fashion.

Thus, the examples above underline that wet and dry or dry and dry assembly methods can be synergistically combined to produce ordered arrays comprising micro‐ and nanoparticles. In addition, having access to single particles is a critical prerequisite to attaining ordered structures, highlighting the challenges, and concurrent limitations when nanoparticles are involved in dry assembly.

## 4 Applications: Printing and Beyond

The archetypal approach to attain spatially ordered arrays of micro‐ and nanoparticles has been through mechanical removal of particles from assembled homogeneous monolayers on surfaces or at the air‐water interface, e.g., the microcontact printing (µcp) technique.^[^ ^223^, ^224^ ^]^ The microcontact printing of particle arrays represents a donor‐acceptor principle, as particles are transferred from the donor surface to another desired substrate. Note that, while this process of transferring particles can be considered dry, the assembled homogeneous layers on the acceptor substrates may not necessarily be obtained without the use of solvents.

An overwhelming diversity of particle transfer particles exists, also within the domain of soft lithography where typically PDMS is exploited as an intermediate actor within the donor‐acceptor principle^[^ ^224^, ^225^, ^226^ ^]^ to attain ordered arrays of colloidal crystals. The various particle transfer techniques clearly delineated in other reports share a common physical mechanism they are based on: the adhesion of the particles on the final printing substrate should surpass the adhesion of the particles on the donor or the intermediate substrate, as is intuitively expected, i.e., one constantly plays a challenging game of balancing the adhesion of the colloidal particles with different substrates or adhesive layers. In general, the adhesion of particles with respective substrates can be tuned via thermal treatment, adsorped molecules, applied pressure, or electrochemically.^[^ ^224^, ^227^ ^]^

**Figure 14:**
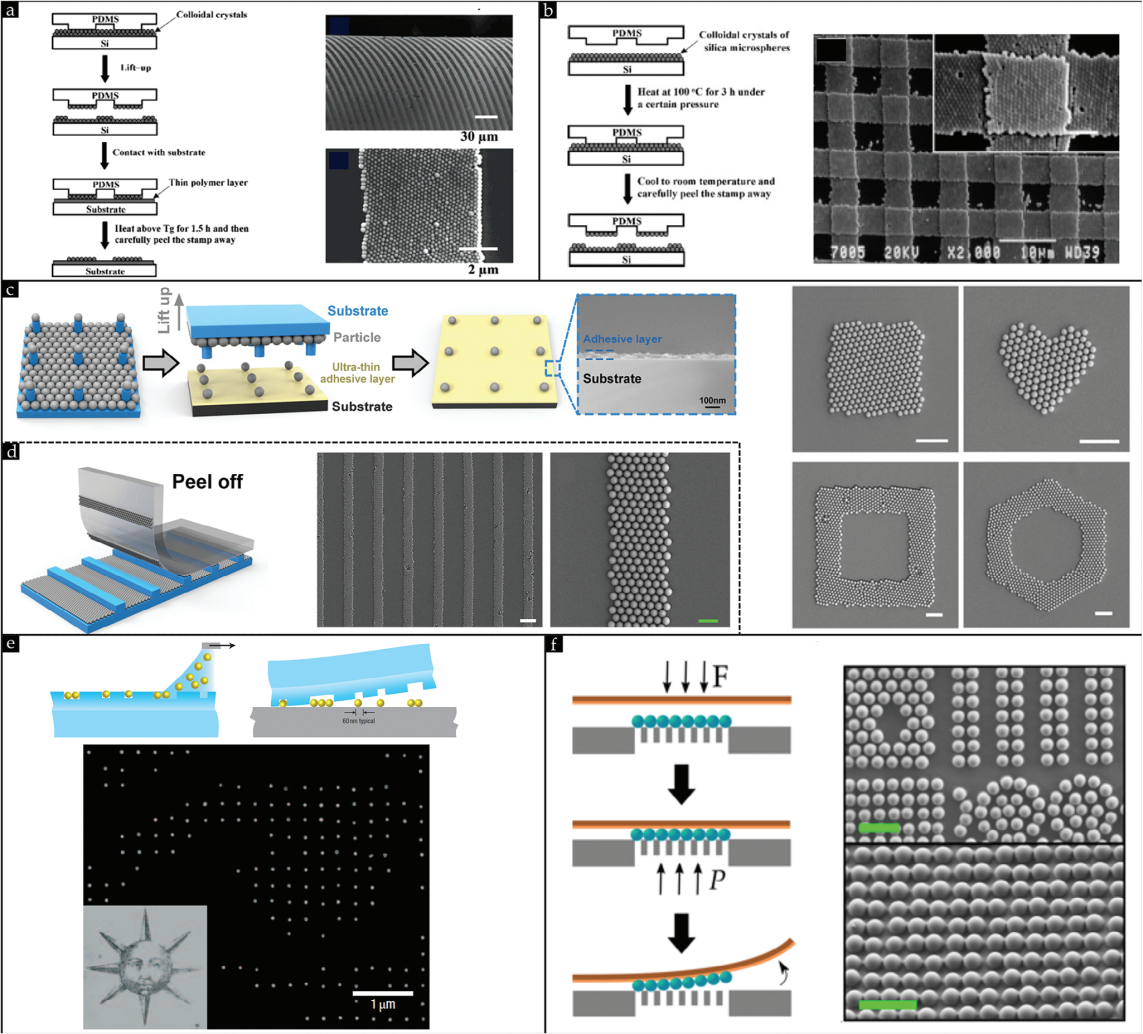
a, left) Schematic illustration of the µcp technique to obtain colloidal crystals patterns. a, right) SEM images of assembled arrays on (top) curved and (bottom) flat. Reproduced with permission.^[^ ^223^ ^]^ Copyright 2004, American Chemical Society. b, left) Schematic representation of the procedure to attain patterned colloidal crystals using the lift‐up soft lithography. b, right) a 3D colloidal crystal obtained using a two‐step lift‐up process (scale bar inset: 2 µm). Reproduced with permission.^[^ ^225^ ^]^ Copyright 2004, Wiley‐VCH. c) Schematic illustration of the nanoparticle transfer printing strategy of micropatterns comprising colloidal crystals on a hard target substrate covered with an ultrathin adhesion layer (inset SEM image). (c, right) SEM images of the corresponding arbitrary 2D micropatterns obtained on a hard substrate (scale bar: 5 µm). d, right) SEM images of the nanoparticle microstripe array on the soft substrate obtained via the (d, left) nanoparticle transfer printing strategy (scale bar: 10 µm (white); 2 µm (green)). (c,d) Reproduced with permission.^[^ ^228^ ^]^ Copyright 2022, Wiley‐VCH. e, top) A self‐assembly process that controls the arrangement of nanoparticles on the printing plate. The entire assembly is printed onto the substrate, whereby the particle positions are precisely retained in high‐resolution. e, bottom) Detail from a printed sun pattern comprising 60‐nm‐diameter Au particles with 280 nm pitch. Reproduced with permission.^[^ ^229^ ^]^ Copyright 2007, Springer Nature. f, left) Schematic representation of microparticle printing process and f, right) corresponding SEM images of assembled ordered arrays from a micro‐structured membrane onto a thin 30:1 PDMS sheet (600 µm) after applying an overpressure *p* (scale bar: 30 µm). Reproduced with permission.^[^ ^213^ ^]^ Copyright 2023, Elsevier.

As can be inferred from **Figure** **14**a–c, ordered assembled crystals (mono‐ or multilayers) and templated substrates are key ingredients to attain the ordered arrays of particles using printing techniques. Typically, substrates with a soft elastomeric character, e.g., PDMS, are utilized as these generate a significant amount of adhesion primarily driven by the contact mechanics force. The protruding areas of the templated substrates lift off ordered monolayers from ordered crystal structures, which are subsequently pressed on, e.g., soft substrates or hard substrates carrying an adhesive layer (cf. Figure 14a–c). The binding force with the adhesive layer can be enhanced by applying a stronger pressure or thermal treatment, as inferred from Figure 14a,b. Note from Figure 14a,b that colloids can also be transferred from multilayer ordered crystal structures, implying that the adhesion among the particles is weaker than the acceptor substrate. The transferring of particles can be on flat surfaces or curved substrates (cf. Figure 14a) or can be used to produce ordered patterned 3D crystal structures using a two‐step lift‐off process of particles (cf. Figure 14b). A soft substrate can also be used by gently peeling off the soft substrate and transferring particles from the protruding surfaces, as displayed in Figure 14d, allowing Li et al.,^[^ ^228^ ^]^ to print micro‐stripes of particles on substrates for flexible electronics, or micropatterns on hard substrates carrying an adhesive layer.

On the other hand, Kraus et al.,^[^ ^229^ ^]^ utilized a so‐called ink‐printing technique, in which the moving meniscus of a nanoparticle suspension positions particles precisely on desired positions inside well‐structures of a templated PDMS substrates, as depicted in Figure 14e. The solvent is rapidly dried to overcome the Brownian motion of the nanoparticles, enabling the transfer of particles from the stamp to another substrate. Consequently, Kraus et al. advanced ink printing to accurately position particles with single‐particle resolution in arbitrary patterns, e.g., a sun with 20 000 single particle spots.

The merit of the dry assembly approaches incorporating perforated silicon devices reported by Van Geite et al. is that the assembled particle arrays can be immediately released by applying an overpressure over the perforated devices and replicated on PDMS substrates, as illustrated in Figure 14f.^[^ ^212^, ^213^ ^]^ As such, this method marks a complete assembly and printing process without using solvents, unlocking the opportunity for producing 3D ordered structures using a layer‐by‐layer assembly strategy.^[^ ^22^ ^]^

**Figure 15:**
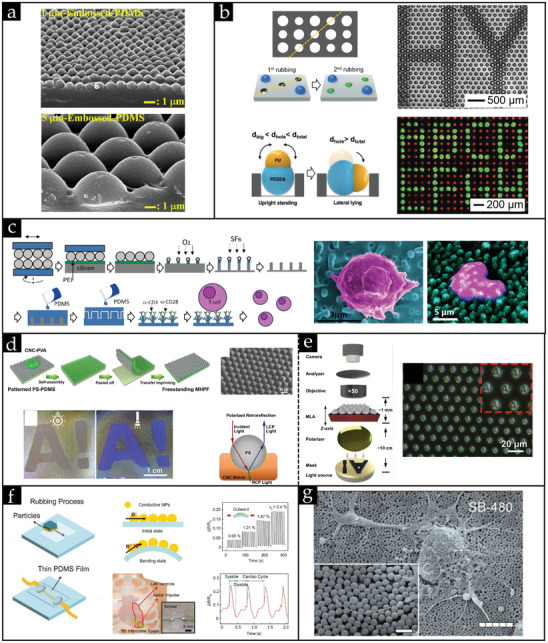
a) Tilted cross sectional SEM images of embossed‐PDMS films. Reproduced with permission.^[^ ^235^ ^]^ Copyright 2016, Elsevier. b, top) Schematic showing the rubbing process used to arrange multiple MPs of different sizes in a template containing holes of corresponding sizes. The bigger MPs are rubbed first, followed by the smaller MPs. Optical microscopy image of a PU template containing multiple MPs of different sizes. b, bottom) Schematic showing the design of hole diameter for upright (standing) and lateral (lying down) configurations of a Janus MP. Fluorescence microscopy image of the correspondingly assembled Janus MPs. Reproduced with permission^[^ ^233^ ^]^ Copyright 2018, Wiley‐VCH. c) Fabrication process flow of the antibody‐functionalized elastic pillars for the activation of T cells. SEM and confocal images of T cells stimulated on the elastic pillars. Reproduced with permission.^[^ ^190^ ^]^ Copyright 2024, American Chemical Society. d) Schematic representation of the transfer imprinting method to prepare a micropatterned photonic film, and (d, top‐right) corresponding SEM image. (d, bottom) Images of 15 µmPS microspheres in a photonic film under indirect diffuse (left) illumination and (right) directional illumination (right), and schematic illustration of the polarization‐sensitive retroreflective. e) Optical imaging performance of polymer film sample as polarization‐sensitive MLAs. (d,e) Reproduced with permission.^[^ ^10^ ^]^ Copyright 2021, Wiley‐VCH. f, left) Schematic representation of the attachment of an ACG sensor to the skin in the region of the 5th intercostal space. An image of the sensor is shown in the inset. 100 nm Au particle powder was rubbed on the pattern to generate 2D percolation of the particles. The PDMS substrate was fully cured and indented with Au nanoparticles by spin coating with diluted PDMS solution. Liquid metal was painted at both ends of the line pattern of the particles, and a conductive fabric was connected. Reproduced with permission.^[^ ^238^ ^]^ Copyright 2016, Wiley‐VCH. (f, right) Relative resistance changes of the ACG sensor during real‐time monitoring of the mechanical movements of the heart. Reproduced with permission.^[^ ^231^ ^]^ Copyright 2019, Wiley‐VCH. g) SEM images of the hippocampal neurons on silica beads of 480 nm in diameter. The scale bar is 5 µm. Insets: Magnified images of filopodial tips. The scale bar is 1 µm. Reproduced with permission.^[^ ^193^ ^]^ Copyright 2012, Wiley‐VCH.

Apart from printing or soft lithography applications, dry assembly approaches have explicitly been utilized to attain ordered structures. As already noted, the simple and fast rubbing method has been predominantly employed to attain monolayers, as showcased in **Figure** **15**, underscoring that ordered particle layers are embedded in diverse applications ranging from optics to surface topography and biomedical.^[^ ^10^, ^230^, ^231^, ^232^, ^233^, ^234^ ^]^ For example, microstructured PDMS structures were replicated (cf. Figure 15a) from an assembled monolayer of PS microparticles. These embossed PDMS surfaces were peeled off from the PS monolayer and utilized as triboelectric nanogenerators (TENGs) that could resist high‐humidity conditions.^[^ ^235^ ^]^

Another adaptation of the rubbing method to attain programmable positioning of two distinct‐sized microparticles on a flexible (elastomeric) template was introduced by Lee et al.,^[^ ^232^ ^]^ As depicted in Figure 15b, the large beads assemble together as they do not fit into the smaller holes, and subsequently, the smaller particles are rubbed. In a similar fashion, the Kim group applied the same concept to assemble anisotropic Janus particles into two different hole sizes,^[^ ^233^ ^]^ attaining two different configurations of the Janus particles (cf. Figure 15b). The accurate positioning of the particles can be readily observed from the fluorescence microscopy images in Figure 15b, enabling colloidal pixel‐based micropatterning technology.

Analogous to the embossing of PDMS surfaces, PDMS nanopillar structures were fabricated by using a silicon mold, as shown in Figure 15c. It is remarkable that here, an automatic rubbing process was developed to attain a monolayer between two PDMS sheets, which was then subsequently transferred on a silicon substrate covered with a PEI layer. This monolayer has been employed as a masking layer reminiscing a colloidal lithography process,^[^ ^18^, ^236^, ^237^ ^]^ followed by additional etching processes to produce the silicon mold, as depicted in Figure 15c. With this nanopatterning technique, Tzadka et al.,^[^ ^190^ ^]^ addressed the limitation of e‐beam lithography for fast and scalable production of elastomer‐based brush arrays for the activation of T cells, allowing to investigation of the proliferation of T cells. The rapid fabrication of such platforms is demanded as immunotherapeutic applications of T cells have garnished significant attention in cancer treatment.

Also, Chu et al.,^[^ ^10^ ^]^ assembled an ordered monolayer of PS microspheres and combined it with a layer of cellulose nanocrystals (CNC) to attain a hierarchical photonic structure or microstructured photonic film, as displayed in Figure 15d. This presents the opportunity for an integrated polarization‐sensitive retroreflector or microlens array (MLA) (cf. Figure 15d,e). The multifunctional photonic devices offer a general framework to manipulate the flow of light within hierarchical architecture.

The Jeong group rubbed dry powder of Au‐colloids, i.e., conductive particles,^[^ ^266^ ^]^ on soft polymer layers to produce skin‐attachable strain sensors.^[^ ^238^ ^]^ They utilized the strain sensor as an apexcardiogram (ACG) to monitor the temporal mechanical motion and hemodynamics of the heart, highlighting the unique opportunities of particle‐based strain sensors (cf. Figure15f). This is a remarkable result, as these types of particles can be brittle, such that they could deform during the rubbing assembly.

At last, in one of the early works on rubbing assembly of monolayers, Kang et al., utilized monolayers of silica colloids to comprehend the developmental responses of neurons to nanostructures (cf. Figure15g), the feature size of which is comparable to that of filopodia (100–300 nm).^[^ ^193^ ^]^

As the advancements of dry rubbing assembly approaches are lagging, their utilization in many application fields is rather limited. Due to its ease, simplicity, and rapidness, the rubbing method has been employed largely, particularly for obtaining ordered monolayer structures. Undoubtedly, when HCP‐ordered particle crystals and micromachining technology become more accessible, the number of applications will also increase. From the above‐mentioned examples, it can be concluded that applications range from particle printing techniques to colloidal lithography to biological sensing applications.

## 5 Conclusions and Future Outlook

In summary, scientists embarked on a journey decades ago to create ordered structures comprising micro‐ and nanoparticles using wet and dry assembly approaches (cf. **Table** **1**), addressing the quest for miniaturized devices. However, assembly methods devoid of solvents remain hardly explored and are an understudied area, such that one may argue that dry assembly is a field that is still in its infancy. The latter is elucidated by the fact that reported studies attempting to gain a profound understanding of the physical phenomena occurring in dry assembly processes at the micro‐ and nanoscale are missing in the literature, hindering the massive development of dry assembly approaches that can neatly manipulate and concurrently balance the dominating surface forces encompassing van der Waals, contact mechanics, capillary, and electrostatic interactions. These interactions can be easily circumvented in solvents, hence the pronounced inclination toward wet assembly at large.

**Table 1:** Comparison of dry and wet assembly methods in terms of various indicators, highlighting challenges and opportunities.

	Dry Assembly	Wet Assembly
Surface Interactions	Capillary, van der Waals,	van der Waals, Electrostatics
	Contact mechanics, Electrostatics (tribocharging)	
Reports	Scarce	Massively studied
Assembled Structures	2D (monolayers & arrays); 3D (scarce)	2D (monolayers & arrays); 3D
Particle supply	Excessive amount of powder	Controlled
Assembly conditions	Humidity, (tribo‐)charging,	Humidity, Solvent & particle properties:
	elasticity of particles and substrates	temperature, surface tension, pH, density
Assembly time	Rapid (<20 s)	Minutes with highly controlled conditions
Versatility & Scalability	Broad spectrum of	Need to be adapted depending
	particle sizes and types on wafer‐scale	on particle size and type, and substrate size.
Smallest Particle size	Rubbing (≈140 ñm), Shaking (3 µm),	A few tens of nanometers
	Electric field (5 µm), Acoustic field (40 µm)	
Particle shapes	Spherical (mostly)	Spherical and shape‐anisotropic
Contamination	Minimal	Prone to contamination/stains of solvents

Nevertheless, a handful of proposed dry assembly methods apply mechanical‐based, electric‐field, laser‐induced, and acoustic‐induced, sometimes only for (sub‐)mm sized spheres, forces to attain 2D‐ordered structures, i.e., monolayers and arrays. The elaborate discussion on these methods clearly underscores the benefits of dry assembly methods compared to wet assembly methods in terms of versatility, scalability, robustness, and rapidness (<20 s). In particular, it is unequivocally shown that in dry assembly similar conditions can be used to assemble a broader spectrum of particles (size and material property) into ordered structures, whereas an excessive amount of dry powder is needed compared to particles in suspensions, marking the drawback of dry assembly methods. From our survey, it became evident that the simple powder rubbing approach has been widely adopted among the proposed methods, albeit mostly on soft elastomeric substrates. Even the automatic rubbing process has been employed between two PDMS sheets. On the other hand, PDMS and the like limit the compatibility of colloidal monolayers in colloidal lithography or sensing and high‐pressure analytical applications that require more inert and robust materials, underpinning the necessity to extend this promising rubbing method to more inert and robust substrates.

Furthermore, the assembly of 3D‐ordered structures without solvents is scarce, posing an immediate opportunity to mature the field. Cementing layers of bricks to build robust structures is commonplace, and perhaps it can inspire us to produce 3D‐ordered structures using a layer‐by‐layer‐assembly strategy,^[^ ^22^ ^]^ i.e., ordered monolayers that can be stacked to produce stable structures. Of course, in this regard, one should also harness the advances in micro‐ and nano‐machining to produce support structures that can aid the dry assembly of 3D‐ordered structures.

In the past decade, we have witnessed an unprecedented revolution in polymer and colloidal chemistry to synthesize particles, which has led to a spectacular variety of building blocks of different shapes, compositions, patterns and functionalities.^[^ ^239^, ^240^, ^241^, ^242^ ^]^ Particles with shape‐anisotropy, e.g., rods, cubes, ellipsoids have attracted great attention, due to higher packing density than spherical particles.^[^ ^239^, ^243^, ^244^, ^245^, ^246^ ^]^ It has also been reported that the shape of particles can also be tuned in a controlled manner by applying ion beam irradiation^[^ ^247^ ^]^ or heat treatment^[^ ^248^ ^]^ on an assembled monolayer of spherical colloids. Despite these advances, the dry assembly of such anisotropic particles, including hematite^[^ ^241^ ^]^ and conductive^[^ ^249^ ^266]^ particles, remain in uncharted area. Note that the different particle geometries will inevitably influence all the surface interaction forces, mainly the contact mechanics force, causing a friction force parallel to the surface, which is often small in spherical particle assembly. Consequently, the attained order of these non‐spherical particles will be affected, as recently observed with cubic silica colloids.^[^ ^189^ ^]^ As inherently, these particles have significantly different responses in electric or magnetic fields than isotropic particles, this propensity can be leveraged to explore the assembly of these particle types without solvents. The assembly of ordered magnetic^[^ ^250^ ^]^ or conductive particles can be relevant to numerous applications, such as robotics, sensing, and electrode materials.^[^ ^249^, ^251^ ^]^ In addition, assembling these novel types of particles is also advantageous in the soft matter field to help us understand crystallization at a smaller scale from a fundamental perspective.

Similar to particles with a shape‐anisotropy, there is much to discover for the assembly of binary particle mixtures on the micro‐ and nanoscale. It was demonstrated by the Whitesides group that millimeter‐sized binary particles can assemble into binary Coulombic crystals using a mechanical shaker.^[^ ^201^ ^]^ However, for smaller beads, this is missing, and other approaches should be explored. For example, (surface) acoustic waves can be applied to agitate smaller particles, both spheres and anisotropic particles and concurrently study their ordering as a result of different interaction forces.

Up to now, we have highlighted the opportunities and challenges that come with the dry assembly of micro‐ and nanoscale particles on surfaces. In order to promote the widespread utilization of assembled particle crystals, also the stability of these monolayers needs to be investigated. The monolayers assembled on soft (polymeric) surfaces, such as PDMS, are stable in air flows and ambient environments.^[^ ^93^, ^99^ ^]^ It becomes more challenging for particles assembled on non‐elastomeric substrates. In ambient environments, the particles are stable, but air flows might disrupt the ordering of the particles depending on the strength of the contact mechanics and electrostatic force.^[^ ^93^ ^]^ And it remains the question of whether any of the assembled monolayers on all these surfaces are stable enough in liquid environments to be useful in pharmaceutical or chemical analysis tools.^[^ ^22^ ^]^ In liquids, the strength of the surface interaction forces changes, and additional forces are present. Especially in droplet‐like configurations, phenomena like buoyancy,^[^ ^252^ ^]^ capillary flows,^[^ ^253^, ^254^ ^]^ Marangoni flows^[^ ^255^ ^]^ and internal flows due to evaporation‐induced contact‐line motion^[^ ^256^ ^]^ come into play, altering the force landscape and thus the stability of the arranged particles.

Furthermore, we envision that AFM and all its associated techniques, e.g., KPFM, will become the workhorse to address the clear impetus in unraveling the surface interaction forces to significantly advance the dry assembly of a large spectrum of colloidal particles into ordered structures. A recent example is the work from Sittl et al.,^[^ ^35^ ^]^ that utilized the colloidal probe technique to study the interaction between sub‐micron rod‐shaped silica particles. Next to these novel experimental studies, we believe that numerical modeling that incorporates all the entangled surface interactions can provide insights into the ordering of colloids.^[^ ^257^, ^258^, ^259^ ^]^ AFM studies may also be of great added value to characterize adhesion among particles and other host surfaces in improving techniques, e.g., polymer brushes,^[^ ^260^ ^]^ to remove excess particles.

At last, roaming around in the dry assembly landscape may feel like a desert, as assembly methods without solvents are scarce. It is unequivocal that we can only take a giant leap in this field by marrying multidisciplinary concepts from chemistry, physics, engineering, and material science. The “dry” natural world still holds many secrets in terms of the micro‐and nanoscale interactions and their concomitant effect on the dry assembly that we need to uncover, such that we can safely conclude at this point that dry assembly methods will plausibly not become prominent in the near future. As such, it remains elusive that solvents (or wet assembly methods) will become obsolete in assembling ordered structures. Moreover, we foresee that dry and wet assembly can work synergistically to at least reduce the amount of solvents significantly, which may be essential in contributing toward the impetus for greener and more sustainable assembly processes.

## Conflict of Interest

The authors declare no conflict of interest.
